# 1,8-Cineole inhibits platelet-leukocyte aggregate formation by reducing P-selectin expression

**DOI:** 10.3389/fphar.2025.1546157

**Published:** 2025-06-05

**Authors:** Julie Petry, Han Mai, Maria Shoykhet, Ali Bashiri Dezfouli, Barbara Wollenberg

**Affiliations:** Department of Otorhinolaryngology, Head and Neck Surgery, School of Medicine and Health, TUM University Hospital, Technical University of Munich, Munich, Germany

**Keywords:** 1,8-cineole, platelets, platelet-leukocyte aggregates, anti-platelet drugs, head and neck squamous cell carcinoma

## Abstract

**Introduction:**

Platelets, traditionally recognized for their role in hemostasis, have increasingly been implicated in cancer progression, including head and neck squamous cell carcinoma (HNSCC). Beyond releasing growth factors and chemokines, platelets modulate leukocyte-mediated proinflammatory responses and effector functions through direct or indirect contact. These processes promote tumor cell proliferation, survival, epithelial to mesenchymal transition (EMT) and extravasation. Consequently, targeting platelet-leukocyte aggregate (PLA) formation represents a promising pharmacological strategy to interfere with platelet-mediated pro-tumorigenic effects. 1,8-cineole, a plant-derived metabolite found in several botanical sources, has shown potent anti-platelet effects through modulation of the adenosine A_2A_ receptor signaling. However, its influence on PLA formation has not been investigated.

**Methods:**

In this study, we analyzed platelet activation and PLA formation in HNSCC patients compared to healthy donors. A co-culture system combined with blocking antibodies was employed to elucidate the mechanisms of PLA formation. Moreover, the pharmacological effects of 1,8-cineole were compared with those of conventional anti-platelet drugs.

**Results:**

The results revealed elevated P-selectin expression and enhanced PLA formation in HNSCC patients. PLA formation was predominantly mediated through P-selectin-PSGL-1 interactions. *Ex vivo* studies demonstrated that 1,8-cineole significantly reduced PLA formation by inhibiting P-selectin expression on platelets. Notably, traditional anti-platelet agents did not significantly inhibit PLA formation, despite effectively reducing platelet aggregation.

**Discussion:**

These findings identify a pharmacological effect of 1,8-cineole in disrupting platelet-leukocyte interactions via suppression of the P-selectin-PSGL-1 axis. This suggests that 1,8-cineole offers potential pharmacological benefits in mitigating platelet-mediated inflammation and tumor progression.

## 1 Introduction

Thrombosis represents a significant complication and a leading cause of mortality among cancer patients (Brose and Lee, 2008; Donnellan et al., 2014; Khorana et al., 2022). It is estimated that up to 30% of all venous thromboembolic events are cancer-related, with metastatic cancers posing an even greater risk (Blom et al., 2006; Bastida and Ordinas, 2009; Timp et al., 2013). The reciprocal relationship between platelets and tumor progression has been recognized for years, yet the mechanisms underpinning this interaction remain incompletely understood. Clarifying these mechanisms is crucial for the development of more effective anti-cancer therapies.

Platelets contribute to cancer progression by releasing cytokines and growth factors such as transforming growth factor β (TGFβ), vascular endothelial growth factor (VEGF), epidermal growth factor (EGF) and platelet-derived growth factor (PDGF), which drive key hallmarks of tumor development, including EMT, angiogenesis, tumor cell migration, and immunosuppression (Takagi et al., 2014; Metelli et al., 2020; Janowska-Wieczorek et al., 2005; Guo et al., 2019; Takemoto et al., 2017; Labelle et al., 2011; Labelle et al., 2014). Notably, thrombocytosis is associated with poor prognostic outcomes across various cancer types (Ikeda et al., 2002; Gücer et al., 1998; Taucher et al., 2003; Kandemir et al., 2005; Yu et al., 2013). Similarly, in HNSCC elevated platelet counts correlate with worse prognosis, and anti-platelet therapies have demonstrated potential in improving clinical outcomes (Chen et al., 2009; Rachidi et al., 2014; Pardo et al., 2017). Despite the well-characterized role of platelets in many cancer entities, their involvement in HNSCC remains insufficiently explored.

The platelet-mediated cancer progression through the release of cytokines and growth factors is dependent on the activation of platelets. In this process, platelets can interact directly with tumor cells or the tumor microenvironment via multiple pathways. For instance, platelets can bind podoplanin on cancer cells via C-type lectin-like immune receptor 2 (CLEC-2) (Rayes et al., 2019). Moreover, tumor cell-derived extracellular vesicles are able to activate platelets via the expression of tissue factor, inducing the intrinsic coagulation pathway (Bastida et al., 1984; Gomes et al., 2017) and several cancer types secrete immunoglobulins, activating platelets via FcγRIIa (Kdimati et al., 2021; Miao et al., 2019). Additionally, tumor-secreted molecules like ADP, thromboxane A2 (TXA_2_) and arachidonic acid (AA) further stimulate platelet activation (Nie et al., 2023; Liao et al., 2022; Morris et al., 2022). Beyond their direct role in tumor biology, platelets significantly influence the tumor microenvironment through interactions with leukocytes, forming platelet-leukocyte aggregates (PLAs). These PLAs serve as hubs for inflammation and immune modulation, amplifying the pro-tumorigenic microenvironment. PLAs promote leukocyte recruitment, activation, extravasation, phenotype switch and changes in effector functions (Kral et al., 2016; Semple et al., 2011; Zuchtriegel et al., 2016). These interactions not only enhance local inflammation but also facilitate tumor invasion and metastasis, promoting an inflammatory milieu conducive to cancer progression (Germano et al., 2008). A critical mechanism by which PLAs drive metastasis is through their promotion of neutrophil extracellular traps (NETs). NETs have been shown to increase the adhesion of tumor cells to lung and liver microvasculature, leading to tumor cell migration and invasion (Albrengues et al., 2018). Additionally, NETs have been implicated in shielding tumor cells from immune attack, further contributing to cancer progression (Ma et al., 2020). PLAs also affect monocyte and macrophage functions by promoting a pro-inflammatory phenotype. Platelet interactions with monocytes can induce the release of inflammatory cytokines such as tumor necrosis factor α (TNFα), interleukin (IL)-1β, IL-6, and IL-8, which reinforce the inflammatory tumor microenvironment (Passacquale et al., 2011; Suzuki et al., 2013). Moreover, PLAs impair the differentiation of monocytes into functionally impaired dendritic cells, thereby hampering antigen uptake as well as impairing antigen cross-presentation and subsequent activation of T cells (Singh et al., 2021; Hilf et al., 2002). Similarly, platelet-T cell aggregates suppress T cell activation, proliferation, and cytokine secretion, thereby facilitating immune evasion by the tumor (Polasky et al., 2020). Through these multifaceted interactions, PLAs exacerbate inflammation and drive processes such as angiogenesis, EMT, and metastasis, all of which are critical hallmarks of cancer progression. PLA formation is not only limited to cancer, but is also observed in several conditions, like cardiovascular diseases, autoinflammatory diseases and infectious diseases (Finsterbusch et al., 2018; Pluta et al., 2022). Given their central role in connecting inflammation, immune modulation and cancer biology, targeting PLAs represents a promising therapeutic avenue to mitigate platelet-mediated inflammation and pro-tumorigenic functions and thus potentially improve cancer outcomes.

Given the critical role of PLAs in exacerbating inflammation and tumor progression, targeting these aggregates may offer therapeutic benefits. In this context, 1,8-cineole, a natural monoterpene found in essential oil extracted from various botanical sources, including Eucalyptus globulus Labill. [Myrtaceae], Rosmarinus officinalis L. [Lamiaceae], or Cinnamomum camphora (L.) J.Presl [Lauraceae], has emerged as a promising candidate (Hoch et al., 2023). Historically, various cultures—including Chinese, Indian, and Indigenous Australian traditions—have incorporated 1,8-cineole into their medicinal practices to address ailments like respiratory and digestive problems, pain, and fever (Chandorkar et al., 2021). 1,8-cineole has proven clinical efficacy on the respiratory tract and has shown therapeutic benefits in inflammatory airway diseases, such as asthma or chronic obstructive pulmonary disease (COPD) (Juergens et al., 2003; Juergens et al., 2020). Furthermore, in several *in vitro* and *in vivo* approaches, 1,8-cineole has demonstrated anti-inflammatory properties (Hoch et al., 2023; Juergens et al., 1998; Juergens et al., 2004). Thus, 1,8-cineole is used in several applications as the main metabolite in both liquid and solid formulations, typically prescribed for upper and lower airway diseases including common cold, asthma, bronchitis, COPD and sinusitis (Hoch et al., 2023). In addition, 1,8-cineole exhibits anti-tumor effects, including inhibition of proliferation, induction of apoptosis, and reduced cell migration in several tumor entities (Murata et al., 2013; Roettger et al., 2017; Rodenak-Kladniew et al., 2020a; Rodenak-Kladniew et al., 2020b). We and others have recently shown that, apart from its anti-inflammatory properties, 1,8-cineole harbors strong anti-platelet activation and aggregation properties (Alatawi et al., 2021; Petry et al., 2024). Specifically, 1,8-cineole induces its antiaggregatory effect via activation of the adenosine A_2A_ receptor, inducing elevated cyclic adenosine monophosphate (cAMP) levels and a protein kinase A (PKA)-mediated anti-platelet effect (Petry et al., 2024). Given its strong anti-inflammatory, anti-tumorigenic, and anti-platelet properties, we hypothesized that 1,8-cineole might effectively suppress PLA formation, thereby mitigating platelet-driven cancer progression. Evaluating its impact on PLAs not only extends our understanding of its therapeutic potential in cancer but also suggests broader implications for inflammatory diseases where PLAs contribute to pathogenesis.

In the present study, we investigated the phenotype of platelets and PLAs in HNSCC patients and explored the impact of 1,8-cineole (CNL-1976^®^) on these aggregates. Specifically, we aimed to unravel the underlying mechanisms driving PLA formation and assess the potential of 1,8-cineole to mitigate this process compared to classical medications known to interfere with platelet function.

## 2 Materials and methods

### 2.1 Chemicals and drugs

1,8-cineole (CNL-1976®) was provided by Klosterfrau Healthcare Group, Cassella-med GmbH & Co. KG (Cologne, Germany) with a purity of 99.5% and stock solutions were prepared by solving native extract in ethanol (5 M) followed by a final dilution in Tyrode buffer (137 mM NaCl, 2 mM KCl, 12 mM NaHCO3, 0.3 mM NaH2PO4, 1 mM MgCl2, 5 mM HEPES, 5.5 mM glucose, 0.5% bovine serum albumin). The platelet agonist, arachidonic acid (AA, #23401) was obtained from Sigma-Aldrich, thrombin receptor activator peptide 6 (TRAP, #3497) from Tocris, convulxin (CVX, #119-02) from Pentapharm, U46619 (#16450) from Cayman Chemical, IV.3 was a kind gift from Ronald Taylor (University of Virginia) and goat-anti mouse F (ab’)_2_ fragment (Fab, #115-006-062) was purchased from Jackson ImmunoResearch. The platelet antagonists, prostaglandin I_2_ (PGI_2_, #P6188) were purchased from Sigma, tirofiban from Hexal, cangrelor (#S3727) from Selleckchem and acetylsalicylic acid (ASA) from Bayer AG.

### 2.2 Whole blood staining

Venous blood was collected into sodium citrate 3.2% (Sarstedt, Germany) from HNSCC patients as well as healthy donors after given informed consent according to the declaration of Helsinki. The study has been approved by the local ethics committee of the Technical University Munich (2020-474_2-S-NP). Whole blood staining was performed immediately after blood draw to evaluate the PLA formation. Hence, 50 µL of blood was incubated with 50 µL of an antibody mixture, consisting of the following antibodies diluted 1:100: APC anti-CD41 (#303710; BioLegend), AF488 anti-CD45 (#304017; BioLegend), PerCP anti-CD3 (#300326; BioLegend), BV421 anti-CD14 (#325628; BioLegend) for 15 min. Subsequently, red blood cell lysis was performed by adding 1 mL of VersaLyse Solution (#A09777, Beckman Coulter), followed by 25 µL of IOTest 10X Concentrate (#A07800, Beckman Coulter) to fix samples. After 20 min of incubation, samples were immediately measured on a Cytoflex S (Beckman Coulter). The gating strategy used to identify the different leukocyte subpopulations is shown in Supplementary Figure S1.

### 2.3 Platelet isolation

Blood from healthy donors was taken in citrate tubes and platelet isolation was performed as previously described (Petry et al., 2024). In brief, platelet rich plasma (PRP) was obtained after centrifugation at 150 rcf for 20 min without brake. To prevent platelet activation, PRP was incubated with 400 nM PGI_2_ and centrifuged at 800 rcf for 10 min without brake. Platelet pellet was resuspended in Tyrode buffer, purity and cell number assessed by flow cytometry and platelet concentration adjusted to 5 × 10^5^ platelets/µL in Tyrode buffer.

### 2.4 Leukocyte isolation

Leukocytes were isolated from remaining blood after platelet isolation using the custom whole blood PBMC isolation kit from Miltenyi (#130-126-359). Remaining blood was diluted 1:2 with PBS and isolation was performed as declared by the manufacturer’s protocol. Leukocyte concentration was assessed using a Neubauer chamber and the concentration adjusted to 1 × 10^6^ cells/mL in RPMI 1640 medium (#72400, Thermo Fisher) supplemented with 10% FCS (#A5256801, Thermo Fisher) and 1% penicillin/streptomycin (#15140, Thermo Fisher).

### 2.5 Platelet-leukocyte aggregate formation

To artificially induce platelet-leukocyte aggregates (PLA) and in order to better understand and analyze the origin of these PLA formations, co-culture assays with leukocytes and platelets in a ratio of 1/50 were performed. To do so, 2 × 10^5^ leukocytes in 200 µL were seeded into a 96-well U-bottom plate and allowed to rest 37°C. 1 × 10^8^ platelets were stimulated with indicated agonists for 5 min. If not stated otherwise, TRAP (10 µM), IV.3 (200 ng/mL) followed by crosslinking with goat-anti-mouse F (ab’)_2_ fragment (7.5 μg/mL), CVX (62.5 ng/mL), U46619 (4.28 µM) and AA (125 µM) were used as platelet agonists. After activation, 1 × 10^7^ platelets were transferred to the leukocytes and co-cultured for 15 min at 37°C. Next, co-culture was fixed with 0.5% paraformaldehyde (PFA) for 15 min at RT, washed with FACS buffer (PBS with 2.5 mM EDTA and 1% FCS). Subsequently, cells were incubated with APC anti-CD41 (1:100, #303710, BioLegend), BV510 anti-CD16 (1:100, #360730, BioLegend), BV421 anti-CD45 (1:100, #304031, BioLegend), PE/Cy7 anti-CD14 (1:100, #325617, BioLegend), PE anti-CD3 (1:100, #300307, BioLegend) and AF488 anti-CD56 (1:100, #318311, BioLegend) for further 15 min, washed twice in FACS buffer and measured via flow cytometry. The gating strategy used to identify the different leukocyte subpopulations is shown in Supplementary Figure S3.

To analyze the effect of 1,8-cineole and other anti-platelet drugs on PLA formation, platelets were pre-incubated with 5 × 10^3^ μM 1,8-cineole or its vehicle control (0.1% v/v ethanol) for 60 min or 50 ng/mL tirofiban, 500 nM cangrelor, 25 mg/mL ASA for 5 min prior being activated with 10 µM TRAP and performing the co-culture assay as described above.

### 2.6 Blocking experiment in co-culture

In order to determine the surface protein responsible for the PLA formation, blocking experiments were performed by using blocking antibodies against known platelet activation markers and integrins. Platelets were activated with TRAP (5 µM) and immediately incubated with 25 μg/mL of the following blocking antibodies for 5 min: Isotype Ctrl (#400107, BioLegend), anti-CD41 (#303702, BioLegend), anti-CD42b (#303902, BioLegend), anti-CD62P (#304902, BioLegend), anti-CD40L (#310802, BioLegend). To block counter parts on leukocytes, cells were pretreated with 25 μg/mL anti-PSGL-1 (#328802, BioLegend) for 5 min. Subsequently, 1 × 10^7^ platelets were transferred to 2 × 10^5^ leukocytes and incubated for 15 min at 37°C. Next, co-culture was fixed with 0.5% PFA for 15 min at RT, washed with FACS buffer, centrifuged for 2 min at 450 rcf and stained with PE anti-CD61 (1:100, #336406, BioLegend), BV421 anti-CD14 (1:100, #325628, BioLegend), APC/Cy7 anti-CD16 (1:100, #360710, BioLegend) for 15 min. Then, cells were washed twice with FACS buffer and measured via flow cytometry. The gating strategy used to identify the different leukocyte subpopulations is shown in Supplementary Figure S4.

### 2.7 Measurement of aggregation

Aggregation was measured with a light transmission aggregometer APACT 4S PLUS (DiaSys Diagnostic Systems GmbH). In brief, 1 × 10^8^ platelets in 200 µL Tyrode buffer or 200 µL PRP (for ADP stimulation) were added to micro cuvettes, stimulated with 50 ng/mL tirofiban, 500 nM cangrelor, 25 mg/mL ASA for 5 min, 5 × 10^3^ μM 1,8-cineole or vehicle control (ethanol 0.1% v/v) for 60 min and a baseline was recorded for 5 min. Platelet aggregation was either induced by adding 10 µM TRAP, 100 µM ADP or 300 ng/mL IV.3 + 15 μg/mL Fab.

### 2.8 Assessment of activation marker

Platelet activation was assessed by measuring the activation marker P-selectin and fibrinogen binding on the cell surface after non-stirring platelet activation. Briefly, 1 × 10^8^ platelets were preincubated with 50 ng/mL tirofiban, 500 nM cangrelor, 25 mg/mL ASA for 5 min or with 5 × 10^3^ μM 1,8-cineole for 60 min at 37°C. Subsequently platelets were activated with 10 µM TRAP for further 10 min at 37°C. Next, 1 × 10^7^ platelets were fixed in 0.5% PFA for at least 15 min. After fixation, platelets were washed with 200 µL FACS buffer, centrifuged for 2 min at 1,200 rcf and stained with APC anti-CD41 (1:100, #303710, BioLegend) and BV510 anti-CD62P (1:100, #304936, BioLegend) for 15 min. Finally, platelets were washed twice with FACS buffer and measured via flow cytometry.

Fibrinogen binding to activated platelets was assessed using AF488-conjugated human fibrinogen (#F13191, Thermo Fisher). Platelets were pretreated with or without 5 × 10^3^ μM 1,8-cineole for 60 min, followed by incubation with 50 μg/mL AF488-fibrinogen. Subsequently platelet activation was induced with 10 µM TRAP for 5 min at 37°C. After activation, platelets were washed with PBS/EDTA, centrifuged at 430 rcf for 5 min, resuspended in FACS buffer and measured via flow cytometry.

### 2.9 Statistical analysis

Each experiment was performed with at least three biological replicates and results are expressed as mean ± standard error of the mean (SEM). All statistical data analyses were conducted using GraphPad Prism 10.1.1. An unpaired two-tailed Student’s t-tests was used to assess the statistical significance between two groups of normally distributed values, while a Mann-Whitney test was used for comparison between healthy donors and HNSCC patients’ samples. Multiple groups were compared using One-way analysis of variance (ANOVA) with Tukey’s *post hoc* test or Dunnett’s *post hoc* test. Statistical significance was considered at *P* < 0.05.

## 3 Results

### 3.1 HNSCC patients showed enhanced circulating platelet-leukocyte aggregate formation and P-selectin expression

**FIGURE 1:**
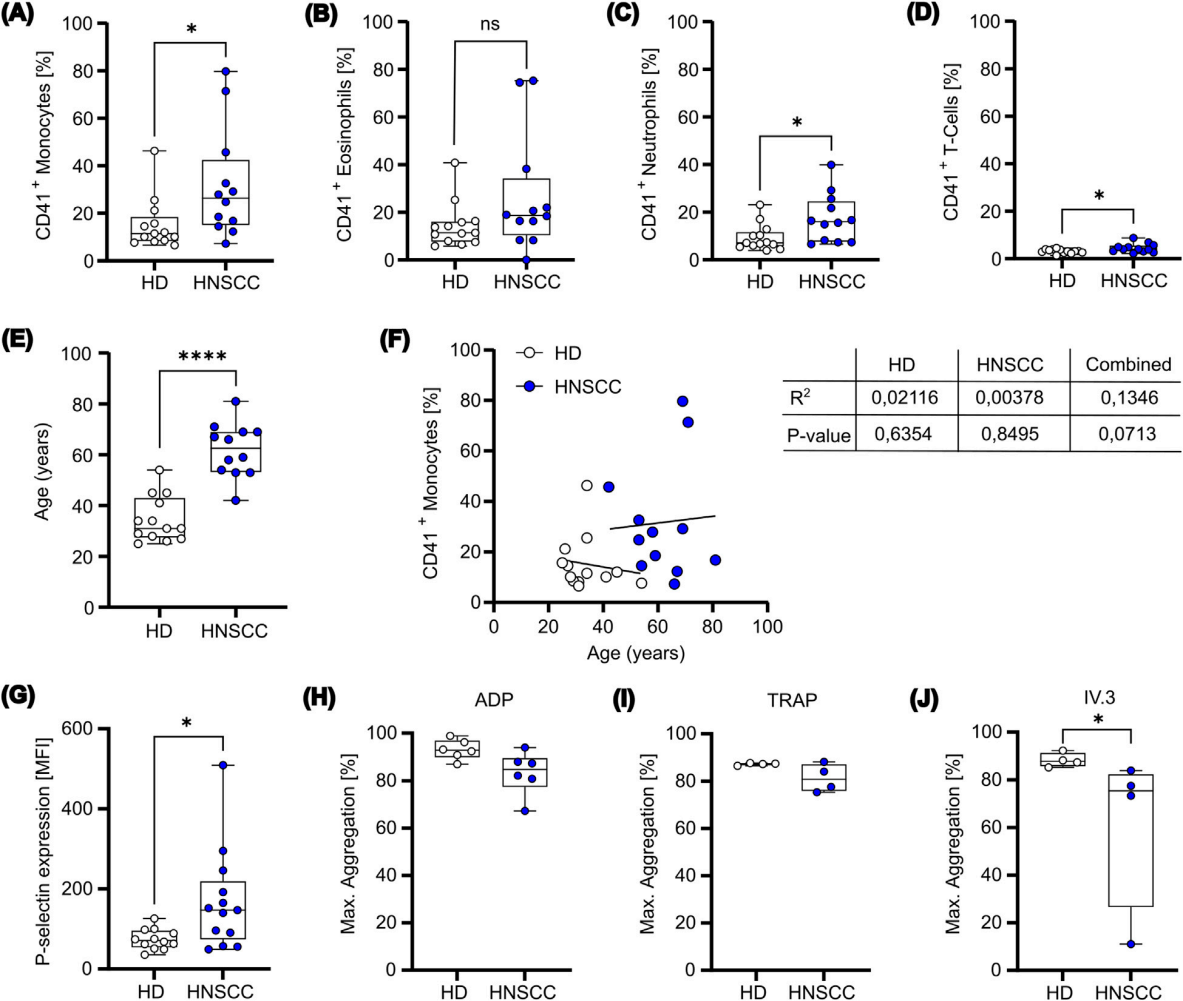
Platelets from HNSCC patients exhibit a prothrombotic phenotype. **(A-D)** Whole blood was collected from healthy donors (n=13) and HNSCC patients (n=12). The samples were immediately stained to determine the prevalence of aggregates between platelets (CD41^+^) and **(A)** monocytes, **(B)** eosinophils, **(C)** neutrophils and **(D)** T-cells. **(E)** Age was compared between HD and HNSCC patients. **(F)** Correlation between CD41^+^ monocytes and age were identified. Data were fitted with a simple linear regression, with the corresponding R^2^ and p-value indicated. **(G)** Platelets isolated from healthy donors and HNSCC patients were stained for P-selectin expression on the cell surface. **(H-J)** HD and HNSCC platelets were stimulated ex vivo with ADP **(H)**, TRAP **(I)** or IV.3+Fab **(J)** for 10 min and maximal aggregation was measured. Data is presented as box plot with mean and minimum to maximum values. *P < 0.05, ****P < 0.0001. Mann-Whitney test was used for comparison between healthy donors and HNSCC patients.

To effectively modulate both innate and adaptive immune responses, platelets establish direct interactions with various leukocyte subtypes, thereby promoting cancer growth and metastasis. Thus, to explore whether these platelet-leukocyte interactions were also observed in HNSCC patients, we first measured circulating PLAs in the whole blood from healthy donors (HD) and HNSCC patients. Leukocyte subtypes were identified in whole blood using the gating strategy illustrated in Supplementary Figure S1. Our findings indicate a marked increase in PLAs across all leukocyte subsets in HNSCC patients. CD14 high monocytes displayed the highest levels of increased platelet aggregates (15.22% ± 10.85% in healthy donors vs. 31.72% ± 22.98% in HNSCC patients), followed by neutrophils (9.27% ± 5.54% vs. 17.42% ± 10.19%) and T cells (3.18% ± 0.84% vs. 4.53% ± 1.87%), whereas eosinophil-platelet aggregates were not significantly altered (14.38% ± 9.49% vs. 26.43% ± 24.42%) (Figures 1A–D). Notably, while platelet-lymphocyte aggregates were significantly increased in HNSCC patients, this subset exhibited the lowest overall percentage of platelet binding compared to other leukocyte subsets. These findings suggest that both healthy and HNSCC-derived platelets show a preference for binding to leukocytes of the innate immune system over those of the adaptive immune system. Given the observed age difference between HD and HNSCC patients (Figure 1E; Supplementary Table S1), we further performed linear regression analyses to evaluate whether age influenced PLA formation. However, monocyte-platelet aggregate formation did not correlate with age, neither within individual groups nor in the combined dataset (Figure 1F). Similar results were found for neutrophil-, eosinophil-, and T-cell-platelet aggregate formation, except for the combined T-cell-platelet aggregate formation, which showed a minor correlation with age (Supplementary Figure S2A–C). Additionally, PLA formation was not affected by the sex of the donor (Supplementary Figure S2D). These results suggest that the observed increase in PLA formation in HNSCC patients is likely attributed to disease-related mechanisms rather than demographic differences. Additionally, a comparison between the basal activation state of single platelets showed a significantly higher level of P-selectin expression on platelets of HNSCC patients than HDs (Figure 1G). A higher basal P-selectin expression often implies a systemic activation of platelets, rendering platelets “exhausted,” which is measured by impaired *ex vivo* reactivity (Baaten et al., 2017). By investigating the reactivity of isolated platelets from both populations upon stimulation with TRAP and ADP, no relevant differences were found (Figures 1H,I). In contrast, platelet activation after crosslinking the low affinity receptor for IgG (FcγRIIa) with the monoclonal antibody IV.3 and Fab fragment, showed slightly decreased reactivity for HNSCC platelets (Figure 1J). The observed unequal distribution of PLAs among leukocyte subsets, together with the slightly increased P-selectin expression suggests that the binding mechanism may depend on platelet activation and on subtype-specific ligands, which are differentially expressed across various leukocyte populations.

### 3.2 Platelet activation is indispensable for PLA formation

**FIGURE 2:**
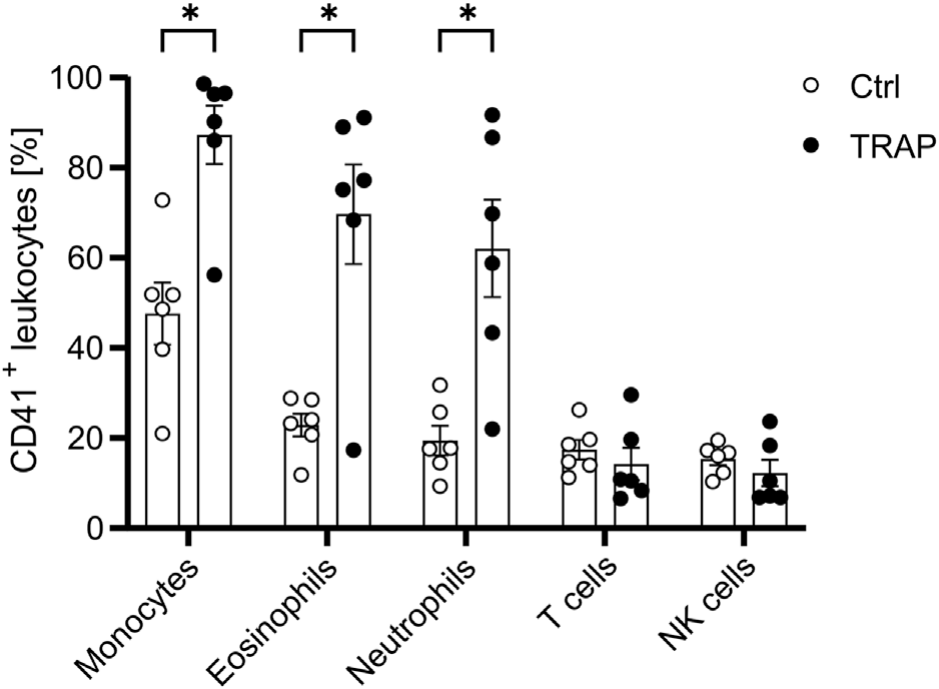
Platelet activation enhances PLA formation. Platelets were either left unstimulated or stimulated with 5 µM TRAP for 5 min before being co-cultured with leukocytes at a ratio of 1:50 for 15 min. The presence of CD41^+^ leukocyte subpopulations was subsequently identified and analyzed via flow cytometry as outlined in Supplementary Figure S3. Data is presented as mean ± SEM. Statistical significance is denoted as *P < 0.05. Unpaired Students’ t-tests were used for comparison between untreated and treated groups.

To better understand the binding mechanism behind these PLAs and identify potential strategies for alleviating PLA formation, a co-culture system with platelets and leukocytes from HDs was established. To this end, resting or activated platelets were co-cultured with leukocytes in a physiological ratio of 50 platelets to 1 leukocyte for 15 min. Leukocyte subtypes were identified using the gating strategy illustrated in Supplementary Figure S3. Importantly, platelet activation with TRAP was essential for promoting PLA formation, with the highest levels observed between activated platelets and CD14 high monocytes, followed by eosinophils and neutrophils. Platelet-T cell and platelet-NK cell aggregates remained relatively low and were not significantly altered (Figure 2). These results align with PLA data from the whole blood of HDs and HNSCC patients (Figures 1A–D). Furthermore, the data indicate that platelet activation is required for PLA formation, suggesting the presence of activation markers as requirement for PLA formation.

### 3.3 PLA formation is dependent on P-selectin expression

**FIGURE 3:**
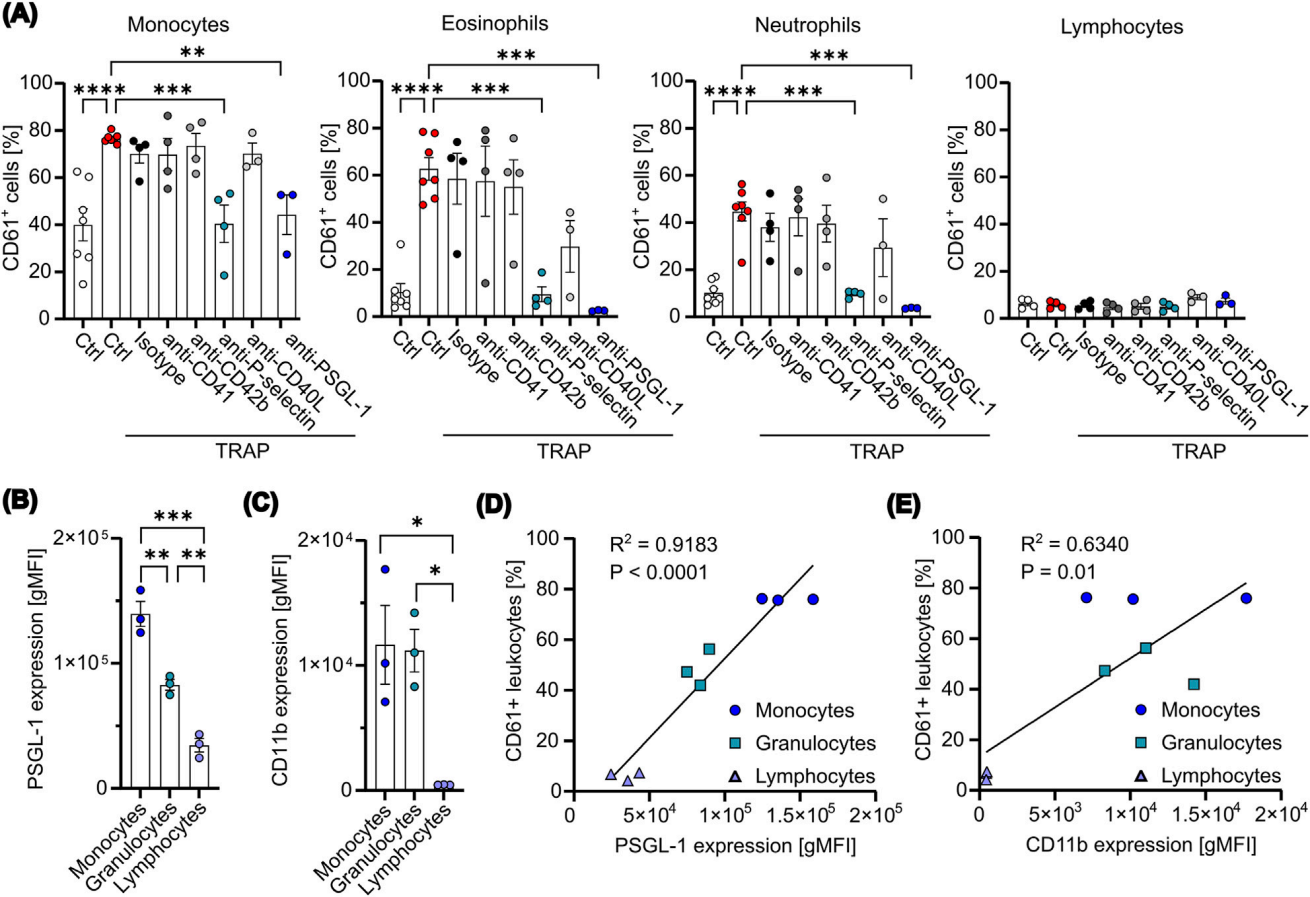
P-selectin expression mediates PLA formation in an in vitro co-culture assay. **(A)** Platelets were pretreated with 5 μM TRAP for 2 min, followed by the addition of the indicated blocking antibodies (10 μg/mL) for additional 5 min. Subsequently, platelets were co-cultured with leukocytes at a ratio of 1:50 for 15 min. For PSGL-1 blockade, leukocytes were pretreated with anti-PSGL-1 (10 μg/mL) for 10 min. PLAs were analyzed via flow cytometry (n=3-7). **(B, C)** The expression levels of PSGL-1 (B) and CD11b **(C)** were measured on the surface of freshly isolated leukocytes using flow cytometry (n=3). **(D, E)** The percentages of CD61+ leukocytes were plotted against the expression levels of PSGL-1 **(D)** or CD11b **(E)**. Data were fitted with a simple linear regression, with the corresponding R2 and p-value (P) indicated (n=3). Data are presented as mean ± SEM. Statistical significance is denoted as *P < 0.05, **P < 0.01, ***P < 0.005, ****P < 0.0001. One-way ANOVA followed by Dunnett’s post-hoc test was used for analysis in **(A)** and Tukey’s post-hoc test for **(B)** and **(C)**.

Next, to identify the ligand expressed on platelets responsible for the underlying PLA formation in our co-culture system, we used several blocking antibodies for common ligands described to play an essential role in PLA formation. Leukocyte subtypes were identified using the gating strategy shown in Supplementary Figure S4. We found that TRAP-mediated PLA formation could be prevented by an antibody targeting P-selectin, whereas inhibition of CD41, CD42b or CD40L did not affect the formation of platelet aggregates with monocytes, neutrophils or eosinophils significantly (Figure 3A). Mechanistically, P-selectin binding glycoprotein ligand 1 (PSGL-1) on leukocytes binds to P-selectin on platelets or endothelial cells. Thus, to confirm the data, we preincubated leukocytes with an anti-PSGL-1 antibody before co-culture. Indeed, by blocking PSGL-1, TRAP-mediated PLA formation was completely abolished (Figure 3A). We therefore concluded that P-selectin is one of the main molecules involved in the formation of PLAs with monocytes, neutrophils and eosinophils in our experimental setting, aligning with our observation that platelet activation is necessary for enhanced PLA formation. Next, to investigate the preferential binding of platelets to monocytes and granulocytes over lymphocytes, we evaluated the expression of PSGL-1 and CD11b, the latter known to bind to CD41 and CD42b, across different leukocyte subsets. Quantification showed that PSGL-1 expression was highest on CD14 high monocytes, followed by granulocytes and lymphocytes, with all differences being statistically significant (Figure 3B). In contrast, CD11b was equally distributed on CD14 high monocytes and granulocytes, while no expression was found on lymphocytes (Figure 3C). Remarkably, the differential expression patterns of PSGL-1 on leukocytes showed a strong and highly significant correlation with the percentages of PLA formation (R^2^ = 0.9183) (Figure 3D). In contrast, a weaker but still significant correlation was observed between CD11b expression and PLA formation (R^2^ = 0.6340) (Figure 3E). However, no correlation was found between CD11b expression and PLA formation in monocytes and granulocytes alone, in contrast to PSGL-1 (Supplementary Figure S5A–B). These results indicate that the P-selectin-PSGL-1 axis is likely the primary mediator of platelets binding to leukocytes, while CD41-or CD42b-CD11b axis may play a supportive role *in vivo*, but appears negligible in our *in vitro* experimental system. Overall, this data implies that targeting the P-selectin-PSGL-1 axis might be effective for modulating PLA formation in a therapeutic context.

### 3.4 1,8-cineole inhibits TRAP-induced formation of PLA

**FIGURE 4:**
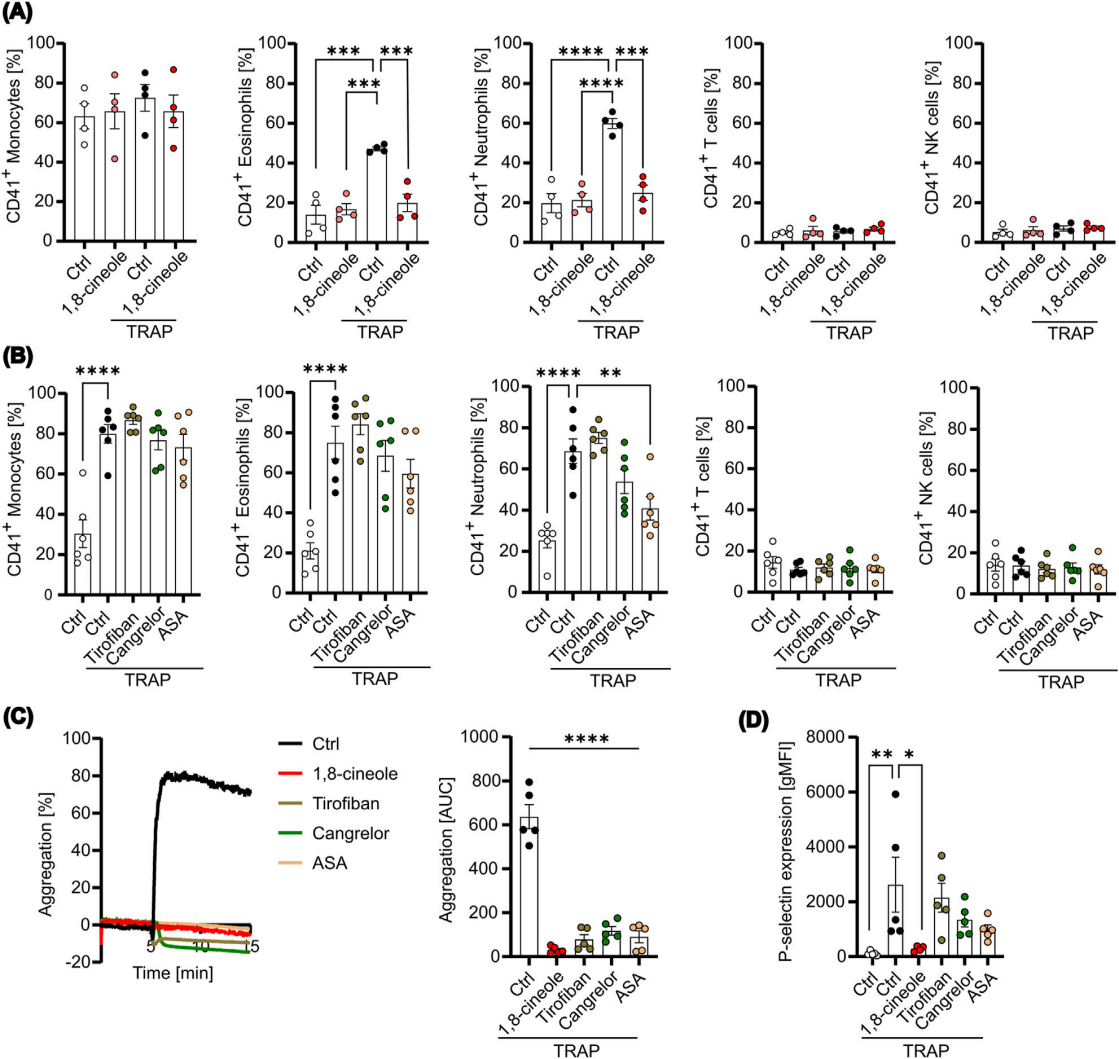
PLA formation is affected by 1,8-cineole. Platelets were pretreated with or without 1,8-cineole or the indicated antithrombotic drugs and subsequently activated with TRAP for 10 min. **(A)** PLA formation was measured with 1,8-cineole-treated platelets after 15 min co-incubation with leukocytes by flow cytometry (n=4). **(B)** PLA formation was determined with antithrombotic-treated platelets after 15 min of co-culture by flow cytometry (n=3). **(C)** Aggregation was determined with treated or untreated platelets for 10 min (n=3). Representative Graph (left panel) and area under the curve [AUC] (right panel) are shown (n=5). **(D)** P-selectin expression on the cell surface was measured. Data are presented as mean ± SEM. *P < 0.05, **P < 0.01, ***P < 0.005, ****P < 0.0001. One-way ANOVA followed by Dunnett’s post-hoc test was used.

PLA formation can aggravate inflammatory diseases, including various cancers, by promoting tumor growth. Since 1,8-cineole (Supplementary Figure S6A) is known not only for its anti-inflammatory and anti-cancer properties, but also for its modulation of platelet activation and aggregation induced by different platelet agonists, we were interested in exploring its influence on PLA formation *ex vivo*. Notably, in our previous work, we showed that 1,8-cineole significantly reduces platelet surface expression of key activation markers, including P-selectin, CD63, CD40L and CD107a (Petry et al., 2024), as well as fibrinogen binding (Supplementary Figure S6B). Given that P-selectin plays a pivotal role in PLA formation, we hypothesized that 1,8-cineole might effectively inhibit PLA formation by suppressing the P-selectin-PSGL-1 interaction. To test this hypothesis, we measured PLA formation with resting or activated platelets after pretreatment with 1,8-cineole. Titration of 1,8-cineole on TRAP-stimulated platelets revealed a concentration-dependent inhibition of platelet aggregation (Supplementary Figure S6C). To ensure complete inhibition in subsequent experiments, a concentration of 5,000 µM was used for all assays involving 1,8-cineole. We observed that TRAP-mediated increases in PLA formation of neutrophils and eosinophils were prevented by 1,8-cineole pretreatment, while 1,8-cineole appeared ineffective for CD14 high monocyte-platelet aggregates (Figure 4A). This is likely due to the substantial expression of PSGL-1 on monocytes compared to neutrophils and eosinophils. In line with previous data, TRAP-activated platelets did not enhance platelet interaction with T cells or NK cells, and thus 1,8-cineole did not affect these PLA formations (Figure 4A). However, platelet activation is not solely dependent on thrombin formation, as it can also be induced by a variety of agonists. Particularly in the tumor microenvironment, other platelet stimulants such as collagen, IgG, AA, and TXA_2_ are present. Hence, we further evaluated the effect of 1,8-cineole on PLA formation with stimulants present in the tumor microenvironment. We found no decrease in platelet-monocyte aggregates after treatment with 1,8-cineole (Supplementary Figure S6A), while platelet-neutrophil and platelet-eosinophil aggregates were significantly reduced in IV.3- and CVX-stimulated platelets (Supplementary Figure S6B–C). In line with previous results, T cell and NK cell aggregates were not significantly affected by 1,8-cineole, except for CVX-stimulated platelet-NK cell aggregates (Supplementary Figure S6D–E). These data suggest that 1,8-cineole effectively reduced platelet-leukocyte aggregates in response to a variety of platelet activation stimulants, likely through the reduction of P-selectin expression.

To further assess the pharmacological effects of anti-platelet treatments on PLA formation, we compared 1,8-cineole with clinically used anti-platelet drugs, including tirofiban, cangrelor and acetylsalicylic acid (ASA). Unlike 1,8-cineole, none of the tested anti-platelet drugs significantly inhibited PLA formation in any of the leukocyte subsets, except for platelet-neutrophil aggregates pretreated with ASA (Figure 4B). However, all anti-platelet drugs, including 1,8-cineole, significantly inhibited platelet aggregation, confirming the appropriate concentrations used and demonstrating their effect in targeting thrombus formation (Figure 4C). In contrast, measurement of P-selectin expression after treatment revealed that while 1,8-cineole completely prevented P-selectin expression, tirofiban, cangrelor and ASA treatment did not inhibit its expression (Figure 4D). These findings indicate that 1,8-cineole may inhibit PLA formation through the reduction of P-selectin, whereas conventional anti-platelet drugs, such as tirofiban, cangrelor and ASA likely fail to prevent PLA formation due to their inability to markedly reduce P-selectin expression following platelet activation. Consequently, 1,8-cineole emerges as a promising metabolite for mitigating platelet-leukocyte interactions, and thus platelet-mediated inflammation and pro-tumorigenic functions.

## 4 Discussion

**FIGURE 5:**
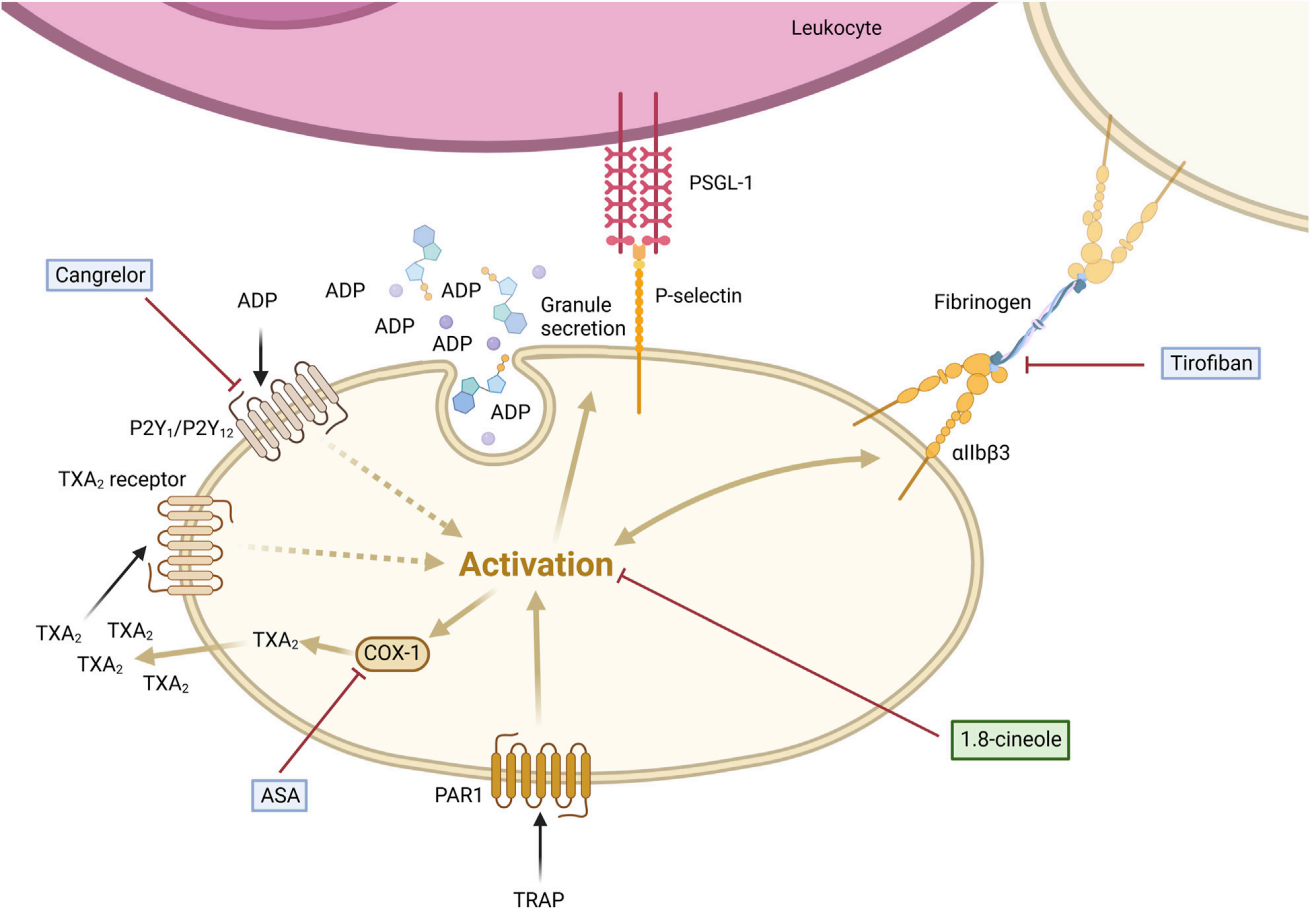
1,8-cineole abrogated TRAP-induced platelet-leukocyte aggregate formation via P-selectin regulation in comparison to classical antithrombotics. PLA formation is induced by the activation of platelets via subsequent P-selectin expression on the cell surface, binding to PSGL-1 expressed on leukocytes. 1,8-cineole inhibits P-selectin expression, thereby reducing PLA formation. Tirofiban blocks the binding of fibrinogen to the GPIIb/IIIa receptor, fully inhibiting aggregation, whereas P-selectin expression and PLA formation remain fully active. Cangrelor antagonizes the ADP receptor, thus inhibiting the platelet activation enhancement, but not the first step of activation. ASA blocks COX-1 activity and subsequent TXA_2_ formation, leading to the inhibition of the platelet activation enhancement, while it does not interfere with the initial step of platelet activation. Created with https://BioRender.com.

This study highlights a significant elevation in P-selectin expression on platelets and increased circulating PLAs in patients with HNSCC compared to HDs. Our findings emphasize that platelet activation is a prerequisite for PLA formation, primarily driven by P-selectin binding to PSGL-1 on leukocytes. Notably, the anti-inflammatory metabolite 1,8-cineole (CNL-1976^®^) reduced PLA formation by suppressing P-selectin expression, demonstrating a superior pharmacological effect compared to classical anti-platelet drugs. This underscores 1,8-cineole’s potential as a targeted disruptor of the P-selectin-PSGL-1 axis, offering a novel approach to mitigate platelet-driven tumor-promoting interactions (Figure 5).

Although platelet-leukocyte interactions have been increasingly studied in various pathological conditions, including cardiovascular diseases and autoimmune disorders, their role in cancer, particularly HNSCC, remains underexplored (Finsterbusch et al., 2018; Joseph et al., 2001). To our knowledge, this study is the first to demonstrate elevated PLAs in HNSCC patients. Similar observations have been reported in a limited number of cancers, such as advanced non-small cell lung cancer (NSCLC) and myeloproliferative disorders (Zamora et al., 2021; Meikle et al., 2020; Jensen et al., 2001). The relatively low prevalence of PLAs in cancer, compared to systemic inflammatory diseases, could be attributed to the locally restricted encounter or infiltration of platelets to the tumor microenvironment, rendering platelet activation and interactions with leukocytes less frequent. In line with these findings, we observed that platelets from HNSCC patients showed marginally increased P-selectin expression, consistent with other reports showing higher P-selectin expression in glioblastoma or hepatocellular carcinoma (Marx et al., 2018; Wang et al., 2019). Of note, high P-selectin expression in the absence of external stimulation, coupled with a diminished response in platelet activation and aggregation following agonist stimulation, is a characteristic of exhausted platelets. This phenotype of circulating hyperactivated yet less reactive platelets has been frequently observed in individuals with various tumor types, as well as in conditions such as sepsis, stroke and COVID-19 (Baaten et al., 2017; Riedl et al., 2017; Tacquard et al., 2023). However, while we found increased P-selectin expression on platelets, indicating a chronic activation and exhausted phenotype, *ex vivo* stimulation of HNSCC platelets with TRAP and ADP preserved their aggregation potential compared to HD platelets. Even though FcγRIIa crosslinking-mediated platelet aggregation in HNSCC patients was marginally lower than in HDs, aggregation was still greater than 70%, suggesting that the platelets are not entirely exhausted. In fact, their increased P-selectin expression points to a primed phenotype that facilitates enhanced interactions with leukocytes. Remarkably, we found that PLA formation in HNSCC patients was lower than in artificial co-culture experiments, likely due to the variability in P-selectin expression levels between *in vivo* and *ex vivo* conditions. Nonetheless, the platelet-monocyte aggregate formation remains a highly sensitive marker of *in vivo* platelet activation, as evidenced by our data, highlighting its potential as a biomarker in cancer (Pluta et al., 2022; Michelson et al., 2001).

Strikingly, activated platelets appeared to exhibit a preference for interacting with leukocytes from the innate immunity (Ahn et al., 2005). This observation suggests that platelets may play a primary role in the rapid recognition and resolution of infections, rather than contributing to the slower processes associated with adaptive immunity. Supporting this hypothesis, our *in vitro* co-culture assays indicated that interactions between P-selectin and PSGL-1 were pivotal for PLA formation. Importantly, PSGL-1 is highly expressed on CD14 high monocytes and granulocytes, while it is only marginally expressed on lymphocytes, and its surface expression strongly correlates with PLA formation. Although we did not observe significant changes in platelet-lymphocyte interactions in our *in vitro* co-culture model, we found a significant increase in PLAs in HNSCC patients. This discrepancy is likely due to the differences between *in vivo* and *in vitro* conditions. In the *in vivo* setting, platelet-leukocyte interactions are likely influenced by the complex array of inflammatory mediators and other factors, which not only promote platelet activation but also facilitate T cell engagement. The critical importance of the P-selectin-PSGL-1 axis in mediating platelet-leukocyte interactions has also been corroborated by other studies (Bournazos et al., 2008; Zarbock et al., 2009). After the initial bond to PSGL-1, firm adhesion is facilitated by interactions between CD11b/CD18 and GPIbα or CD41 via fibrinogen (Wang et al., 2017; Ehlers et al., 2003). Additionally, platelet CD40L mediates physical interaction and signaling with immune cells expressing CD40 (Cognasse et al., 2022). However, these additional interactions were not found to be significantly regulated in our *in vitro* experimental setup.

Comparative analysis of 1,8-cineole with classical anti-platelet drugs revealed its unique ability to inhibit both platelet activation and PLA formation. Particular, we found that 1,8-cineole significantly blocks platelet-neutrophil and platelet-eosinophil interactions, independent of the platelet stimulation agent used. This inhibition of PLA formation likely results from 1,8-cineole´s suppression of P-selectin expression, thereby disrupting the P-selectin-PSGL-1 axis. Noteworthy, classical agents such as tirofiban, cangrelor, and ASA showed minimal impact on P-selectin expression, and consequently, had no effect on PLA formation. This aligns with existing reports, which suggest that anti-platelet drugs primarily target platelet aggregation rather than activation or degranulation. For instance, while tirofiban effectively inhibits platelet aggregation, it significantly enhances the surface expression of P-selectin and the release of CCL5 (Aguiar et al., 2022). Moreover, multiple studies have demonstrated that GPIIb/IIIa antagonists have little to no effect on P-selectin expression following platelet activation, with agents like tirofiban being recognized as weak inhibitors of platelet release reactions (Caron et al., 2002; Barlage et al., 2002; Dickfeld et al., 2001). Furthermore, the anti-platelet effect of ASA is primarily attributed to its inhibition of dense granule release (Rinder et al., 1993). In accordance, ASA has shown only a minor inhibitory effect on the surface expression of P-selectin and CD40L (Parker et al., 2020; Moshfegh et al., 2000), and several studies have concluded that ASA intake does not significantly alter P-selectin expression or PLA formations (Zhao et al., 2001; Storey et al., 2002; Li et al., 2003; Klinkhardt et al., 2003). Besides GPIIb/IIIa and COX-1 inhibitors, the P2Y_12_ antagonist ticagrelor has shown only a slight effect on P-selectin expression in a COVID-19 model (Schrottmaier et al., 2021), while clopidogrel was reported to only marginally reduce TRAP-induced P-selectin expression and PLA formations (Xiao and Théroux, 2004). These findings demonstrate that PLAs are partially and unspecifically targeted by current antithrombotic regimens. It is tempting to speculate that the clinical use of anti-platelet drugs may impact the hemostatic roles of platelets, but not the immunological and tumorigenic roles of platelets, as these processes often rely on P-selectin-PSGL-1 interactions (Semple et al., 2011). In contrast, the phosphodiesterase 3 inhibitor cilostazol effectively interfered with the interaction between platelets and leukocytes by reducing P-selectin expression on the platelet surface (Ito et al., 2004). Similarly, the prostacyclin analogue illoprost and the adenylyl cyclase (AC) agonist forskolin prevented P-selectin upregulation and abrogated platelet-neutrophil aggregate formations (Konstantopoulos et al., 1998; Libersan et al., 2003). Unlike tirofiban, cangrelor or ASA, these compounds are cAMP-elevating agents, similar to 1,8-cineole, and demonstrate broader anti-platelet effects by inhibiting both aggregation and activation (Petry et al., 2024). This dual-action mechanism positions 1,8-cineole as a promising candidate for targeting platelet-mediated inflammatory and tumor-promoting processes. While various other phytochemicals have been studied for their anti-platelet effects, their influence on PLA formation remains largely unexamined (Tamer et al., 2022). Most phytochemicals tested for anti-platelet activity are dietary metabolites rather than clinically applied agents. In contrast, 1,8-cineole, which is already used in medical practice, exhibits not only anti-platelet but also anti-inflammatory and anti-cancer properties, making it a promising candidate for targeting platelet-driven inflammation in cancer and other diseases (Hoch et al., 2023). Although the small, lipophilic nature of 1,8-cineole raises the possibility of assay interference, our previous work demonstrated a highly specific mode of action via the adenosine A_2A_ receptor pathway (Petry et al., 2024). Using a broad panel of pharmacological inhibitors, only those targeting this pathway were able to reverse the inhibitory effects on platelet aggregation, strongly arguing against non-specific activity or pan-assay interference compound-like behavior.

Particularly, P-selectin has emerged as a therapeutic target in various diseases, including cardiovascular conditions, systemic lupus erythematosus, and sickle cell anemia (Ataga et al., 2017; Scherlinger et al., 2021; Schmitt et al., 2015). In oncology, P-selectin inhibition has been associated with reduced tumor growth and improved survival in preclinical models of melanoma and glioblastoma (Muz et al., 2015; Yeini et al., 2021). Knockout studies of platelet molecules further refine the notion that platelets and particular P-selectin, play a crucial role in tumor metastasis (Kim et al., 1998). Our findings support the growing evidence that targeting P-selectin can disrupt platelet-promoting tumor progression, offering new avenues for therapeutic interventions in HNSCC and potentially other cancers. To fully establish the therapeutic potential of 1,8-cineole in cancer treatment, further studies are required to determine its optimal concentration and dosing regimen. Reported effective concentrations of 1,8-cineole *in vitro* vary widely, ranging from micromolar to millimolar levels, depending on the tumor cell type (Hoch et al., 2023). *In vivo* studies have demonstrated pharmacological effects at a dose of 50 mg/kg when administered subcutaneously in mice (Murata et al., 2013). Additionally, previous research utilizing 1,8-cineole in inflammatory murine models has employed oral doses ranging from 10 to 120 mg/kg (Zhao et al., 2014; Li et al., 2016). However, the most suitable route of administration for cancer treatment remains to be clarified. Given that 1,8-cineole is predominantly administered orally in existing formulations, this route may be the first choice for further evaluation. Regarding its toxicity profile, studies have reported an oral acute LD50 of 3,849 mg/kg body weight in mice, with repeated doses of up to 1,500 mg/kg over 50 days being well tolerated (Xu et al., 2014). Importantly, we have previously demonstrated that 1,8-cineole does not exhibit cytotoxic effects on platelets at concentrations up to 25 mM (Petry et al., 2024). These findings suggest a favorable safety profile, but further investigations are necessary to assess the metabolite’s long-term effects and therapeutic index in cancer models.

Despite the robust findings regarding 1,8-cineole and other anti-platelet drugs in inhibiting PLA formation and their potential use in tumor disease, the study has several limitations. First, the effects of 1,8-cineole on PLA formation were evaluated using *ex vivo* methods. While informative, these conditions may not fully replicate the complex dynamics of the tumor microenvironment *in vivo*. Validation in animal models and further clinical trials are necessary to confirm the translational relevance. Additionally, while we observed increased P-selectin expression, elevated PLAs in HNSCC patients, and a potent reduction of P-selectin expression and PLA formation following 1,8-cineole treatment, the study did not explore whether these reductions in PLA formation or P-selectin expression correlate with clinical outcomes, such as tumor progression, response to therapy, or survival. Establishing such clinical correlations would strengthen the significance of the findings. Moreover, although our study examined the association between PLA formation, age, and sex, we did not assess other potential biological factors such as body mass index, smoking status, or platelet counts, which could influence PLA formation. Nevertheless, several studies have reported no significant correlation between PLA formation and these factors, suggesting that their impact may be minimal in certain contexts (Meikle et al., 2020; Gilstad et al., 2009; Liu et al., 2019; Schulte et al., 2024). Additionally, the variability in platelet activation across different patients, potentially influenced by individual tumor characteristics, comorbidities, or medication usage, may introduce variability in the results. Nonetheless, future studies assessing a larger patient cohort could further elucidate whether demographic or biological characteristics modulate PLA formation in HNSCC. Second, this study focused exclusively on circulating PLAs without assessing their presence or functional role within the tumor microenvironment. Investigating PLAs and the impact of 1,8-cineole within tumor tissue could provide deeper insights into their contribution to tumor progression and immune modulation. Third, the potential for 1,8-cineole to synergize with existing treatments, such as chemotherapy or immunotherapy, also remains an open question and warrants further investigation. Nevertheless, we present compelling evidence regarding the activity of 1,8-cineole on PLA formation and we believe that our study provides valuable insights into the role of PLA in HNSCC, offering a promising foundation for future research and therapeutic strategies targeting platelet-mediated mechanisms in cancer.

Future investigations are needed to fully characterize the role of 1,8-cineole on platelets and their tumorigenic effects. It is of particular interest to not only elucidate whether 1,8-cineole inhibits PLA formation, but also whether it reduces platelet-driven tumor progression. In particular, determining whether the pharmacological effect of 1,8-cineole in inhibiting PLA formation *in vitro* are also applicable *in vivo* will be crucial. In this regard, future studies should involve cancer mouse models, particularly HNSCC, to assess the combined effects of 1,8-cineole on platelet activation, tumor cells, and platelet-induced inflammation and tumorigenesis. Expanding the research to include other cancer types, especially those with increased PLA formation, will help determine whether the observed effects are cancer-specific or more broadly applicable.

## 5 Conclusion

This study highlights the importance of innovative approaches to target platelet activation and PLAs in cancer treatment. 1,8-cineole might represent a novel anti-aggregatory metabolite with significant potential to disrupt PLA formation and thus to mitigate platelet-mediated tumor progression. Unlike classical anti-platelet drugs that primarily target platelet aggregation, 1,8-cineole specifically disrupts the P-selectin-PSGL-1 axis, addressing the inflammatory and immune-modulating aspects of cancer progression. While 1,8-cineole shows promise, further research is required to validate its clinical efficacy and safety in cancer therapy. Nonetheless, given its ability to modulate critical platelet-leukocyte interactions, 1,8-cineole could pave the way for novel therapeutic strategies aimed at reducing the pro-tumorigenic functions of platelets in HNSCC and other cancers.

## Data Availability

The raw data supporting the conclusions of this article will be made available by the authors, without undue reservation.

## References

[B1] Aguiar B. M. Idel C. Wollenberg B. Mannhalter C. Verschoor A. (2022). Tirofiban potentiates agonist-induced platelet activation and degranulation, despite effectively inhibiting aggregation. Platelets 33 (8), 1192–1198. 10.1080/09537104.2022.2078489 35701857

[B2] Ahn K. C. Jun A. J. Pawar P. Jadhav S. Napier S. McCarty O. J. (2005). Preferential binding of platelets to monocytes over neutrophils under flow. Biochem. Biophys. Res. Commun. 329 (1), 345–355. 10.1016/j.bbrc.2005.01.146 15721313

[B3] Alatawi K. A. Ravishankar D. Patra P. H. Bye A. P. Stainer A. R. Patel K. (2021). 1,8-Cineole affects agonists-induced platelet activation, thrombus formation and haemostasis. Cells 10 (10), 2616. 10.3390/cells10102616 34685597 PMC8533741

[B4] Albrengues J. Shields M. A. Ng D. Park C. G. Ambrico A. Poindexter M. E. (2018). Neutrophil extracellular traps produced during inflammation awaken dormant cancer cells in mice. Science 361 (6409), eaao4227. 10.1126/science.aao4227 30262472 PMC6777850

[B5] Ataga K. I. Kutlar A. Kanter J. Liles D. Cancado R. Friedrisch J. (2017). Crizanlizumab for the prevention of pain crises in sickle cell disease. N. Engl. J. Med. 376 (5), 429–439. 10.1056/NEJMoa1611770 27959701 PMC5481200

[B6] Baaten C. Ten Cate H. van der Meijden P. E. J. Heemskerk J. W. M. (2017). Platelet populations and priming in hematological diseases. Blood Rev. 31 (6), 389–399. 10.1016/j.blre.2017.07.004 28756877

[B7] Barlage S. Wimmer A. Pfeiffer A. Rothe G. Schmitz G. (2002). MK-383 (Tirofiban) induces a GPIIb/IIIa receptor conformation which differs from the resting and activated receptor. Platelets 13, 133–140. 10.1080/09533710022149377 12180495

[B8] Bastida E. Ordinas A. (2009). Platelet contribution to the formation of metastatic foci: the role of cancer cell-induced platelet activation. Pathophysiol. Haemostasis Thromb. 18 (1), 29–36. 10.1159/000215780 3047021

[B9] Bastida E. Ordinas A. Escolar G. Jamieson G. A. (1984). Tissue factor in microvesicles shed from U87MG human glioblastoma cells induces coagulation, platelet aggregation, and thrombogenesis. Blood 64 (1), 177–184. 10.1182/blood.v64.1.177.177 6733271

[B10] Blom J. W. Vanderschoot J. P. Oostindiër M. J. Osanto S. van der Meer F. J. Rosendaal F. R. (2006). Incidence of venous thrombosis in a large cohort of 66,329 cancer patients: results of a record linkage study. J. Thromb. Haemost. 4 (3), 529–535. 10.1111/j.1538-7836.2006.01804.x 16460435

[B11] Bournazos S. Rennie J. Hart S. P. Fox K. A. Dransfield I. (2008). Monocyte functional responsiveness after PSGL-1-mediated platelet adhesion is dependent on platelet activation status. Arterioscler. Thromb. Vasc. Biol. 28 (8), 1491–1498. 10.1161/ATVBAHA.108.167601 18497306

[B12] Brose K. M. Lee A. Y. (2008). Cancer-associated thrombosis: prevention and treatment. Curr. Oncol. 15 (Suppl. 1), S58–S67. 10.3747/co.2008.177 18231650 PMC2216419

[B13] Caron A. Théorêt J. F. Mousa S. A. Merhi Y. (2002). Anti-platelet effects of GPIIb/IIIa and P-selectin antagonism, platelet activation, and binding to neutrophils. J. Cardiovasc Pharmacol. 40 (2), 296–306. 10.1097/00005344-200208000-00015 12131559

[B14] Chandorkar N. Tambe S. Amin P. Madankar C. (2021). A systematic and comprehensive review on current understanding of the pharmacological actions, molecular mechanisms, and clinical implications of the genus Eucalyptus. Phytomed. Plus 1 (4), 100089. 10.1016/j.phyplu.2021.100089

[B15] Chen M. H. Chang P. M. Chen P. M. Tzeng C. H. Chu P. Y. Chang S. Y. (2009). Prognostic significance of a pretreatment hematologic profile in patients with head and neck cancer. J. Cancer Res. Clin. Oncol. 135 (12), 1783–1790. 10.1007/s00432-009-0625-1 19551407 PMC11844790

[B16] Cognasse F. Duchez A. C. Audoux E. Ebermeyer T. Arthaud C. A. Prier A. (2022). Platelets as key factors in inflammation: focus on CD40L/CD40. Front. Immunol. 13, 825892. 10.3389/fimmu.2022.825892 35185916 PMC8850464

[B17] Dickfeld T. Ruf A. Pogatsa-Murray G. Müller I. Engelmann B. Taubitz W. (2001). Differential antiplatelet effects of various glycoprotein IIb-IIIa antagonists. Thromb. Res. 101 (2), 53–64. 10.1016/s0049-3848(00)00385-6 11342206

[B18] Donnellan E. Kevane B. Bird B. R. Ainle F. N. (2014). Cancer and venous thromboembolic disease: from molecular mechanisms to clinical management. Curr. Oncol. 21 (3), 134–143. 10.3747/co.21.1864 24940094 PMC4059798

[B19] Ehlers R. Ustinov V. Chen Z. Zhang X. Rao R. Luscinskas F. W. (2003). Targeting platelet-leukocyte interactions: identification of the integrin Mac-1 binding site for the platelet counter receptor glycoprotein Ibalpha. J. Exp. Med. 198 (7), 1077–1088. 10.1084/jem.20022181 14530377 PMC2194217

[B20] Finsterbusch M. Schrottmaier W. C. Kral-Pointner J. B. Salzmann M. Assinger A. (2018). Measuring and interpreting platelet-leukocyte aggregates. Platelets 29 (7), 677–685. 10.1080/09537104.2018.1430358 29461910 PMC6178087

[B21] Germano G. Allavena P. Mantovani A. (2008). Cytokines as a key component of cancer-related inflammation. Cytokine 43 (3), 374–379. 10.1016/j.cyto.2008.07.014 18701317

[B22] Gilstad J. R. Gurbel P. A. Andersen R. E. (2009). Relationship between age and platelet activation in patients with stable and unstable angina. Arch. Gerontol. Geriatr. 48 (2), 155–159. 10.1016/j.archger.2007.12.006 18282622

[B23] Gomes F. G. Sandim V. Almeida V. H. Rondon A. M. R. Succar B. B. Hottz E. D. (2017). Breast-cancer extracellular vesicles induce platelet activation and aggregation by tissue factor-independent and -dependent mechanisms. Thromb. Res. 159, 24–32. 10.1016/j.thromres.2017.09.019 28950217

[B24] Gücer F. Moser F. Tamussino K. Reich O. Haas J. Arikan G. (1998). Thrombocytosis as a prognostic factor in endometrial carcinoma. Gynecol. Oncol. 70 (2), 210–214. 10.1006/gyno.1998.5078 9740692

[B25] Guo Y. Cui W. Pei Y. Xu D. (2019). Platelets promote invasion and induce epithelial to mesenchymal transition in ovarian cancer cells by TGF-β signaling pathway. Gynecol. Oncol. 153 (3), 639–650. 10.1016/j.ygyno.2019.02.026 30928020

[B26] Hilf N. Singh-Jasuja H. Schwarzmaier P. Gouttefangeas C. Rammensee H. G. Schild H. (2002). Human platelets express heat shock protein receptors and regulate dendritic cell maturation. Blood 99 (10), 3676–3682. 10.1182/blood.v99.10.3676 11986223

[B27] Hoch C. C. Petry J. Griesbaum L. Weiser T. Werner K. Ploch M. (2023). 1,8-cineole (eucalyptol): a versatile phytochemical with therapeutic applications across multiple diseases. Biomed. Pharmacother. 167, 115467. 10.1016/j.biopha.2023.115467 37696087

[B28] Ikeda M. Furukawa H. Imamura H. Shimizu J. Ishida H. Masutani S. (2002). Poor prognosis associated with thrombocytosis in patients with gastric cancer. Ann. Surg. Oncol. 9 (3), 287–291. 10.1007/BF02573067 11923136

[B29] Ito H. Miyakoda G. Mori T. (2004). Cilostazol inhibits platelet-leukocyte interaction by suppression of platelet activation. Platelets 15 (5), 293–301. 10.1080/09537100410001715583 15370100

[B30] Janowska-Wieczorek A. Wysoczynski M. Kijowski J. Marquez-Curtis L. Machalinski B. Ratajczak J. (2005). Microvesicles derived from activated platelets induce metastasis and angiogenesis in lung cancer. Int. J. Cancer 113 (5), 752–760. 10.1002/ijc.20657 15499615

[B31] Jensen M. K. de Nully Brown P. Lund B. V. Nielsen O. J. Hasselbalch H. C. (2001). Increased circulating platelet-leukocyte aggregates in myeloproliferative disorders is correlated to previous thrombosis, platelet activation and platelet count. Eur. J. Haematol. 66 (3), 143–151. 10.1034/j.1600-0609.2001.00359.x 11350482

[B32] Joseph J. E. Harrison P. Mackie I. J. Isenberg D. A. Machin S. J. (2001). Increased circulating platelet-leucocyte complexes and platelet activation in patients with antiphospholipid syndrome, systemic lupus erythematosus and rheumatoid arthritis. Br. J. Haematol. 115 (2), 451–459. 10.1046/j.1365-2141.2001.03101.x 11703349

[B33] Juergens L. J. Worth H. Juergens U. R. (2020). New perspectives for mucolytic, anti-inflammatory and adjunctive therapy with 1,8-cineole in COPD and asthma: review on the new therapeutic approach. Adv. Ther. 37 (5), 1737–1753. 10.1007/s12325-020-01279-0 32200535 PMC7467491

[B34] Juergens U. R. Dethlefsen U. Steinkamp G. Gillissen A. Repges R. Vetter H. (2003). Anti-inflammatory activity of 1.8-cineol (eucalyptol) in bronchial asthma: a double-blind placebo-controlled trial. Respir. Med. 97 (3), 250–256. 10.1053/rmed.2003.1432 12645832

[B35] Juergens U. R. Engelen T. Racke K. Stober M. Gillissen A. Vetter H. (2004). Inhibitory activity of 1,8-cineol (eucalyptol) on cytokine production in cultured human lymphocytes and monocytes. Pulm. Pharmacol. Ther. 17 (5), 281–287. 10.1016/j.pupt.2004.06.002 15477123

[B36] Juergens U. R. Stober M. Vetter H. (1998). Inhibition of cytokine production and arachidonic acid metabolism by eucalyptol (1.8-cineole) in human blood monocytes *in vitro* . Eur. J. Med. Res. 3 (11), 508–510. 9810029

[B37] Kandemir E. G. Mayadagli A. Karagoz B. Bilgi O. Turken O. Yaylaci M. (2005). Prognostic significance of thrombocytosis in node-negative colon cancer. J. Int. Med. Res. 33 (2), 228–235. 10.1177/147323000503300211 15790135

[B38] Kdimati S. Mullins C. S. Linnebacher M. (2021). Cancer-cell-derived IgG and its potential role in tumor development. Int. J. Mol. Sci. 22 (21), 11597. 10.3390/ijms222111597 34769026 PMC8583861

[B39] Khorana A. A. Mackman N. Falanga A. Pabinger I. Noble S. Ageno W. (2022). Cancer-associated venous thromboembolism. Nat. Rev. Dis. Prim. 8 (1), 11. 10.1038/s41572-022-00336-y 35177631

[B40] Kim Y. J. Borsig L. Varki N. M. Varki A. (1998). P-selectin deficiency attenuates tumor growth and metastasis. Proc. Natl. Acad. Sci. U. S. A. 95 (16), 9325–9330. 10.1073/pnas.95.16.9325 9689079 PMC21337

[B41] Klinkhardt U. Bauersachs R. Adams J. Graff J. Lindhoff-Last E. Harder S. (2003). Clopidogrel but not aspirin reduces P-selectin expression and formation of platelet-leukocyte aggregates in patients with atherosclerotic vascular disease. Clin. Pharmacol. Ther. 73 (3), 232–241. 10.1067/mcp.2003.13 12621388

[B42] Konstantopoulos K. Neelamegham S. Burns A. R. Hentzen E. Kansas G. S. Snapp K. R. (1998). Venous levels of shear support neutrophil-platelet adhesion and neutrophil aggregation in blood via P-selectin and beta2-integrin. Circulation 98 (9), 873–882. 10.1161/01.cir.98.9.873 9738642

[B43] Kral J. B. Schrottmaier W. C. Salzmann M. Assinger A. (2016). Platelet interaction with innate immune cells. Transfus. Med. Hemother 43 (2), 78–88. 10.1159/000444807 27226790 PMC4872052

[B44] Labelle M. Begum S. Hynes R. O. (2011). Direct signaling between platelets and cancer cells induces an epithelial-mesenchymal-like transition and promotes metastasis. Cancer Cell 20 (5), 576–590. 10.1016/j.ccr.2011.09.009 22094253 PMC3487108

[B45] Labelle M. Begum S. Hynes R. O. (2014). Platelets guide the formation of early metastatic niches. Proc. Natl. Acad. Sci. U. S. A. 111 (30), E3053–E3061. 10.1073/pnas.1411082111 25024172 PMC4121772

[B46] Li N. Hu H. Hjemdahl P. (2003). Aspirin treatment does not attenuate platelet or leukocyte activation as monitored by whole blood flow cytometry. Thromb. Res. 111 (3), 165–170. 10.1016/j.thromres.2003.08.026 14678815

[B47] Li Y. Lai Y. Wang Y. Liu N. Zhang F. Xu P. (2016). 1, 8-cineol protect against influenza-virus-induced pneumonia in mice. Inflammation 39 (4), 1582–1593. 10.1007/s10753-016-0394-3 27351430

[B48] Liao P. Wang W. Wang W. Kryczek I. Li X. Bian Y. (2022). CD8+ T cells and fatty acids orchestrate tumor ferroptosis and immunity via ACSL4. Cancer Cell 40 (4), 365–378.e6. 10.1016/j.ccell.2022.02.003 35216678 PMC9007863

[B49] Libersan D. Rousseau G. Merhi Y. (2003). Differential regulation of P-selectin expression by protein kinase A and protein kinase G in thrombin-stimulated human platelets. Thromb. Haemost. 89 (2), 310–317. 10.1055/s-0037-1613448 12574812

[B50] Liu C. Yang Y. Du L. Chen S. Zhang J. Zhang C. (2019). Platelet-leukocyte aggregate is associated with adverse events after surgical intervention for rheumatic heart disease. Sci. Rep. 9 (1), 13069. 10.1038/s41598-019-49253-3 31506454 PMC6737193

[B51] Ma Y. Wei J. He W. Ren J. (2020). Neutrophil extracellular traps in cancer. MedComm 5 (8), e647. 10.1002/mco2.647 PMC11247337 39015554

[B52] Marx S. Splittstöhser M. Kinnen F. Moritz E. Joseph C. Paul S. (2018). Platelet activation parameters and platelet-leucocyte-conjugate formation in glioblastoma multiforme patients. Oncotarget 9 (40), 25860–25876. 10.18632/oncotarget.25395 29899827 PMC5995223

[B53] Meikle C. K. Meisler A. J. Bird C. M. Jeffries J. A. Azeem N. Garg P. (2020). Platelet-T cell aggregates in lung cancer patients: implications for thrombosis. PLoS One 15 (8), e0236966. 10.1371/journal.pone.0236966 32776968 PMC7416940

[B54] Metelli A. Wu B. X. Riesenberg B. Guglietta S. Huck J. D. Mills C. (2020). Thrombin contributes to cancer immune evasion via proteolysis of platelet-bound GARP to activate LTGF-β. Sci. Transl. Med. 12 (525), eaay4860. 10.1126/scitranslmed.aay4860 31915300 PMC7814995

[B55] Miao S. Shu D. Zhu Y. Lu M. Zhang Q. Pei Y. (2019). Cancer cell-derived immunoglobulin G activates platelets by binding to platelet FcγRIIa. Cell Death Dis. 10 (2), 87. 10.1038/s41419-019-1367-x 30692520 PMC6349849

[B56] Michelson A. D. Barnard M. R. Krueger L. A. Valeri C. R. Furman M. I. (2001). Circulating monocyte-platelet aggregates are a more sensitive marker of *in vivo* platelet activation than platelet surface P-selectin: studies in baboons, human coronary intervention, and human acute myocardial infarction. Circulation 104 (13), 1533–1537. 10.1161/hc3801.095588 11571248

[B57] Morris K. Schnoor B. Papa A.-L. (2022). Platelet cancer cell interplay as a new therapeutic target. Biochim Biophys Acta Rev Cancer. 1877 (5), 188770. 10.1016/j.bbcan.2022.188770 35926688

[B58] Moshfegh K. Redondo M. Julmy F. Wuillemin W. A. Gebauer M. U. Haeberli A. (2000). Antiplatelet effects of clopidogrel compared with aspirin after myocardial infarction: enhanced inhibitory effects of combination therapy. J. Am. Coll. Cardiol. 36 (3), 699–705. 10.1016/s0735-1097(00)00817-2 10987587

[B59] Murata S. Shiragami R. Kosugi C. Tezuka T. Yamazaki M. Hirano A. (2013). Antitumor effect of 1, 8-cineole against colon cancer. Oncol. Rep. 30 (6), 2647–2652. 10.3892/or.2013.2763 24085263

[B60] Muz B. Azab F. de la Puente P. Rollins S. Alvarez R. Kawar Z. (2015). Inhibition of P-selectin and PSGL-1 using humanized monoclonal antibodies increases the sensitivity of multiple myeloma cells to bortezomib. Biomed. Res. Int. 2015, 417586. 10.1155/2015/417586 26539491 PMC4619821

[B61] Nie J. Z. Wang M. T. Nie D. (2023). Regulations of tumor microenvironment by prostaglandins. Cancers (Basel) 15 (12), 3090. 10.3390/cancers15123090 37370700 PMC10296267

[B62] Pardo L. Valero C. López M. García J. Camacho M. Quer M. (2017). The prognostic value of pretreatment platelet count in patients with head and neck squamous cell carcinoma. Auris Nasus Larynx 44 (3), 313–318. 10.1016/j.anl.2016.06.009 27401121

[B63] Parker W. A. E. Schulte C. Barwari T. Phoenix F. Pearson S. M. Mayr M. (2020). Aspirin, clopidogrel and prasugrel monotherapy in patients with type 2 diabetes mellitus: a double-blind randomised controlled trial of the effects on thrombotic markers and microRNA levels. Cardiovasc Diabetol. 19 (1), 3. 10.1186/s12933-019-0981-3 31910903 PMC6945631

[B64] Passacquale G. Vamadevan P. Pereira L. Hamid C. Corrigall V. Ferro A. (2011). Monocyte-platelet interaction induces a pro-inflammatory phenotype in circulating monocytes. PLoS One 6 (10), e25595. 10.1371/journal.pone.0025595 22022418 PMC3192052

[B65] Petry J. Weiser T. Griesbaum L. Schröder K. Hoch C. C. Bashiri Dezfouli A. (2024). 1.8-cineole prevents platelet activation and aggregation by activating the cAMP pathway via the adenosine A(2A) receptor. Life Sci. 350, 122746. 10.1016/j.lfs.2024.122746 38810792

[B66] Pluta K. Porębska K. Urbanowicz T. Gąsecka A. Olasińska-Wiśniewska A. Targoński R. (2022). Platelet-Leucocyte aggregates as novel biomarkers in cardiovascular diseases. Biol. (Basel). 11 (2), 224. 10.3390/biology11020224 PMC8869671 35205091

[B67] Polasky C. Wendt F. Pries R. Wollenberg B. (2020). Platelet induced functional alteration of CD4(+) and CD8(+) T cells in HNSCC. Int. J. Mol. Sci. 21 (20), 7507. 10.3390/ijms21207507 33053760 PMC7588893

[B68] Rachidi S. Wallace K. Day T. A. Alberg A. J. Li Z. (2014). Lower circulating platelet counts and antiplatelet therapy independently predict better outcomes in patients with head and neck squamous cell carcinoma. J. Hematol. Oncol. 7, 65. 10.1186/s13045-014-0065-5 25260646 PMC4189675

[B69] Rayes J. Watson S. P. Nieswandt B. (2019). Functional significance of the platelet immune receptors GPVI and CLEC-2. J. Clin. Invest 129 (1), 12–23. 10.1172/JCI122955 30601137 PMC6307936

[B70] Riedl J. Kaider A. Marosi C. Prager G. W. Eichelberger B. Assinger A. (2017). Decreased platelet reactivity in patients with cancer is associated with high risk of venous thromboembolism and poor prognosis. Thromb. Haemost. 117 (1), 90–98. 10.1160/TH16-02-0123 27761580 PMC6522348

[B71] Rinder C. S. Student L. A. Bonan J. L. Rinder H. M. Smith B. R. (1993). Aspirin does not inhibit adenosine diphosphate-induced platelet alpha-granule release. Blood 82 (2), 505–512. 10.1182/blood.v82.2.505.505 7687162

[B72] Rodenak-Kladniew B. Castro A. Stärkel P. Galle M. Crespo R. (2020a). 1,8-Cineole promotes G0/G1 cell cycle arrest and oxidative stress-induced senescence in HepG2 cells and sensitizes cells to anti-senescence drugs. Life Sci. 243, 117271. 10.1016/j.lfs.2020.117271 31926243

[B73] Rodenak-Kladniew B. Castro M. A. Crespo R. Galle M. García de Bravo M. (2020b). Anti-cancer mechanisms of linalool and 1,8-cineole in non-small cell lung cancer A549 cells. Heliyon 6 (12), e05639. 10.1016/j.heliyon.2020.e05639 33367122 PMC7749389

[B74] Roettger A. Bruchhage K. L. Drenckhan M. Ploetze-Martin K. Pries R. Wollenberg B. (2017). Inhibitory effect of 1,8-cineol on beta-catenin regulation, WNT11 expression, and cellular progression in HNSCC. Front. Oncol. 7, 92. 10.3389/fonc.2017.00092 28589081 PMC5438970

[B75] Scherlinger M. Guillotin V. Douchet I. Vacher P. Boizard-Moracchini A. Guegan J. P. (2021). Selectins impair regulatory T cell function and contribute to systemic lupus erythematosus pathogenesis. Sci. Transl. Med. 13 (600), eabi4994. 10.1126/scitranslmed.abi4994 34193612

[B76] Schmitt C. Abt M. Ciorciaro C. Kling D. Jamois C. Schick E. (2015). First-in-Man study with inclacumab, a human monoclonal antibody against P-selectin. J. Cardiovasc Pharmacol. 65 (6), 611–619. 10.1097/FJC.0000000000000233 25714598 PMC4461388

[B77] Schrottmaier W. C. Pirabe A. Pereyra D. Heber S. Hackl H. Schmuckenschlager A. (2021). Platelets and antiplatelet medication in COVID-19-related thrombotic complications. Front. Cardiovasc Med. 8, 802566. 10.3389/fcvm.2021.802566 35141292 PMC8818754

[B78] Schulte C. Pieper L. Frye M. Waldeyer C. Neumann J. T. Brunner F. J. (2024). Antiplatelet drugs do not protect from platelet-leukocyte aggregation in coronary artery disease. J. Thromb. Haemostasis 22 (2), 553–557. 10.1016/j.jtha.2023.04.041 37225020

[B79] Semple J. W. Italiano J. E. Jr. Freedman J. (2011). Platelets and the immune continuum. Nat. Rev. Immunol. 11 (4), 264–274. 10.1038/nri2956 21436837

[B80] Singh M. V. Suwunnakorn S. Simpson S. R. Weber E. A. Singh V. B. Kalinski P. (2021). Monocytes complexed to platelets differentiate into functionally deficient dendritic cells. J. Leukoc. Biol. 109 (4), 807–820. 10.1002/JLB.3A0620-460RR 32663904 PMC7854860

[B81] Storey R. F. Judge H. M. Wilcox R. G. Heptinstall S. (2002). Inhibition of ADP-induced P-selectin expression and platelet-leukocyte conjugate formation by clopidogrel and the P2Y12 receptor antagonist AR-C69931MX but not aspirin. Thromb. Haemost. 88 (3), 488–494. 10.1055/s-0037-1613242 12353080

[B82] Suzuki J. Hamada E. Shodai T. Kamoshida G. Kudo S. Itoh S. (2013). Cytokine secretion from human monocytes potentiated by P-selectin-mediated cell adhesion. Int. Arch. Allergy Immunol. 160 (2), 152–160. 10.1159/000339857 23018521

[B83] Tacquard C. Mouriaux C. Delabranche X. Bourdon C. Eckly A. Magnenat S. (2023). Platelet dysfunction and thrombus instability in flow conditions in patients with severe COVID-19. Thromb. Res. 221, 137–148. 10.1016/j.thromres.2022.11.004 36376109 PMC9642035

[B84] Takagi S. Takemoto A. Takami M. Oh-Hara T. Fujita N. (2014). Platelets promote osteosarcoma cell growth through activation of the platelet-derived growth factor receptor-Akt signaling axis. Cancer Sci. 105 (8), 983–988. 10.1111/cas.12464 24974736 PMC4317862

[B85] Takemoto A. Okitaka M. Takagi S. Takami M. Sato S. Nishio M. (2017). A critical role of platelet TGF-β release in podoplanin-mediated tumour invasion and metastasis. Sci. Rep. 7 (1), 42186. 10.1038/srep42186 28176852 PMC5297242

[B86] Tamer F. Tullemans B. M. E. Kuijpers M. J. E. Claushuis T. A. M. Heemskerk J. W. M. (2022). Nutrition phytochemicals affecting platelet signaling and responsiveness: implications for thrombosis and hemostasis. Thromb. Haemost. 122 (6), 879–894. 10.1055/a-1683-5599 34715717

[B87] Taucher S. Salat A. Gnant M. Kwasny W. Mlineritsch B. Menzel R. C. (2003). Impact of pretreatment thrombocytosis on survival in primary breast cancer. Thromb. Haemost. 89 (6), 1098–1106. 10.1055/s-0037-1613413 12783124

[B88] Timp J. F. Braekkan S. K. Versteeg H. H. Cannegieter S. C. (2013). Epidemiology of cancer-associated venous thrombosis. Blood 122 (10), 1712–1723. 10.1182/blood-2013-04-460121 23908465

[B89] Wang B. Zhu J. Ma X. Wang H. Qiu S. Pan B. (2019). Platelet activation status in the diagnosis and postoperative prognosis of hepatocellular carcinoma. Clin. Chim. Acta 495, 191–197. 10.1016/j.cca.2019.03.1634 30946815

[B90] Wang Y. Gao H. Shi C. Erhardt P. W. Pavlovsky A. Soloviev D A. (2017). Leukocyte integrin Mac-1 regulates thrombosis via interaction with platelet GPIbα. Nat. Commun. 8 (1), 15559. 10.1038/ncomms15559 28555620 PMC5477519

[B91] Xiao Z. Théroux P. (2004). Clopidogrel inhibits platelet-leukocyte interactions and thrombin receptor agonist peptide-induced platelet activation in patients with an acute coronary syndrome. J. Am. Coll. Cardiol. 43 (11), 1982–1988. 10.1016/j.jacc.2003.10.071 15172401

[B92] Xu J. Hu Z. Q. Wang C. Yin Z. Q. Wei Q. Zhou L. J. (2014). Acute and subacute toxicity study of 1,8-cineole in mice. Int. J. Clin. Exp. Pathol. 7 (4), 1495–1501. 24817945 PMC4014229

[B93] Yeini E. Ofek P. Pozzi S. Albeck N. Ben-Shushan D. Tiram G. (2021). P-selectin axis plays a key role in microglia immunophenotype and glioblastoma progression. Nat. Commun. 12 (1), 1912. 10.1038/s41467-021-22186-0 33771989 PMC7997963

[B94] Yu D. Liu B. Zhang L. Du K. (2013). Platelet count predicts prognosis in operable non-small cell lung cancer. Exp. Ther. Med. 5 (5), 1351–1354. 10.3892/etm.2013.1003 23737877 PMC3671769

[B95] Zamora C. Riudavets M. Anguera G. Alserawan L. Sullivan I. Barba A. (2021). Circulating leukocyte-platelet complexes as a predictive biomarker for the development of immune-related adverse events in advanced non-small cell lung cancer patients receiving anti-PD-(L)1 blocking agents. Cancer Immunol. Immunother. 70 (6), 1691–1704. 10.1007/s00262-020-02793-4 33388994 PMC10991171

[B96] Zarbock A. Müller H. Kuwano Y. Ley K. (2009). PSGL-1-dependent myeloid leukocyte activation. J. Leukoc. Biol. 86 (5), 1119–1124. 10.1189/jlb.0209117 19703898

[B97] Zhao C. Sun J. Fang C. Tang F. (2014). 1,8-cineol attenuates LPS-induced acute pulmonary inflammation in mice. Inflammation 37 (2), 566–572. 10.1007/s10753-013-9770-4 24197825

[B98] Zhao L. Bath P. Heptinstall S. (2001). Effects of combining three different antiplatelet agents on platelets and leukocytes in whole blood *in vitro* . Br. J. Pharmacol. 134 (2), 353–358. 10.1038/sj.bjp.0704248 11564653 PMC1572950

[B99] Zuchtriegel G. Uhl B. Puhr-Westerheide D. Pörnbacher M. Lauber K. Krombach F. (2016). Platelets guide leukocytes to their sites of extravasation. PLoS Biol. 14 (5), e1002459. 10.1371/journal.pbio.1002459 27152726 PMC4859536

