# Correction: 1,8-Cineole inhibits platelet-leukocyte aggregate formation by reducing P-selectin expression

**DOI:** 10.3389/fphar.2025.1656591

**Published:** 2025-07-10

**Authors:** Julie Petry, Han Mai, Maria Shoykhet, Ali Bashiri Dezfouli, Barbara Wollenberg

**Affiliations:** Department of Otorhinolaryngology, Head and Neck Surgery, School of Medicine and Health, TUM University Hospital, Technical University of Munich, Munich, Germany

**Keywords:** 1,8-cineole, platelets, platelet-leukocyte aggregates, anti-platelet drugs, head and neck squamous cell carcinoma

File **Supplementary file 2** was erroneously published with the original version of this paper. The file has now been removed.

**Supplementary file 2** was erroneously cited in the manuscript as “Supplementary Material.” With the removal of **Supplementary file 2**, the corresponding in-text citation has also been deleted.

A correction has been made to the section 2 Materials and methods, *2.1 Chemicals and drugs*, Paragraph 1. The sentence previously stated:

“1,8-cineole (CNL-1976^®^) was provided by Klosterfrau Healthcare Group, Cassella-med GmbH & Co. KG (Cologne, Germany) with a purity of 99.5% (**Supplementary Material**) and stock solutions were prepared by solving native extract in ethanol (5 M) followed by a final dilution in Tyrode buffer (137 mM NaCl, 2 mM KCl, 12 mM NaHCO_3_, 0.3 mM NaH_2_PO_4_, 1 mM MgCl_2_, 5 mM HEPES, 5.5 mM glucose, 0.5% bovine serum albumin).”

This has been updated to:

“1,8-cineole (CNL-1976^®^) was provided by Klosterfrau Healthcare Group, Cassella-med GmbH & Co. KG (Cologne, Germany) with a purity of 99.5% and stock solutions were prepared by solving native extract in ethanol (5 M) followed by a final dilution in Tyrode buffer (137 mM NaCl, 2 mM KCl, 12 mM NaHCO_3_, 0.3 mM NaH_2_PO_4_, 1 mM MgCl_2_, 5 mM HEPES, 5.5 mM glucose, 0.5% bovine serum albumin).”

The original version of this article has been updated.

