# Supplementary material for: 1,8-Cineole inhibits platelet-leukocyte aggregate formation by reducing P-selectin expression
Source: Front Pharmacol. 2025 Jun 5;16:1546157. doi: 10.3389/fphar.2025.1546157 (PMC12176828; doi:10.3389/fphar.2025.1546157)
Supplement: Supplementary file 1 [file Supplementaryfile1.docx]

Supplementary Material

# Supplementary Figures


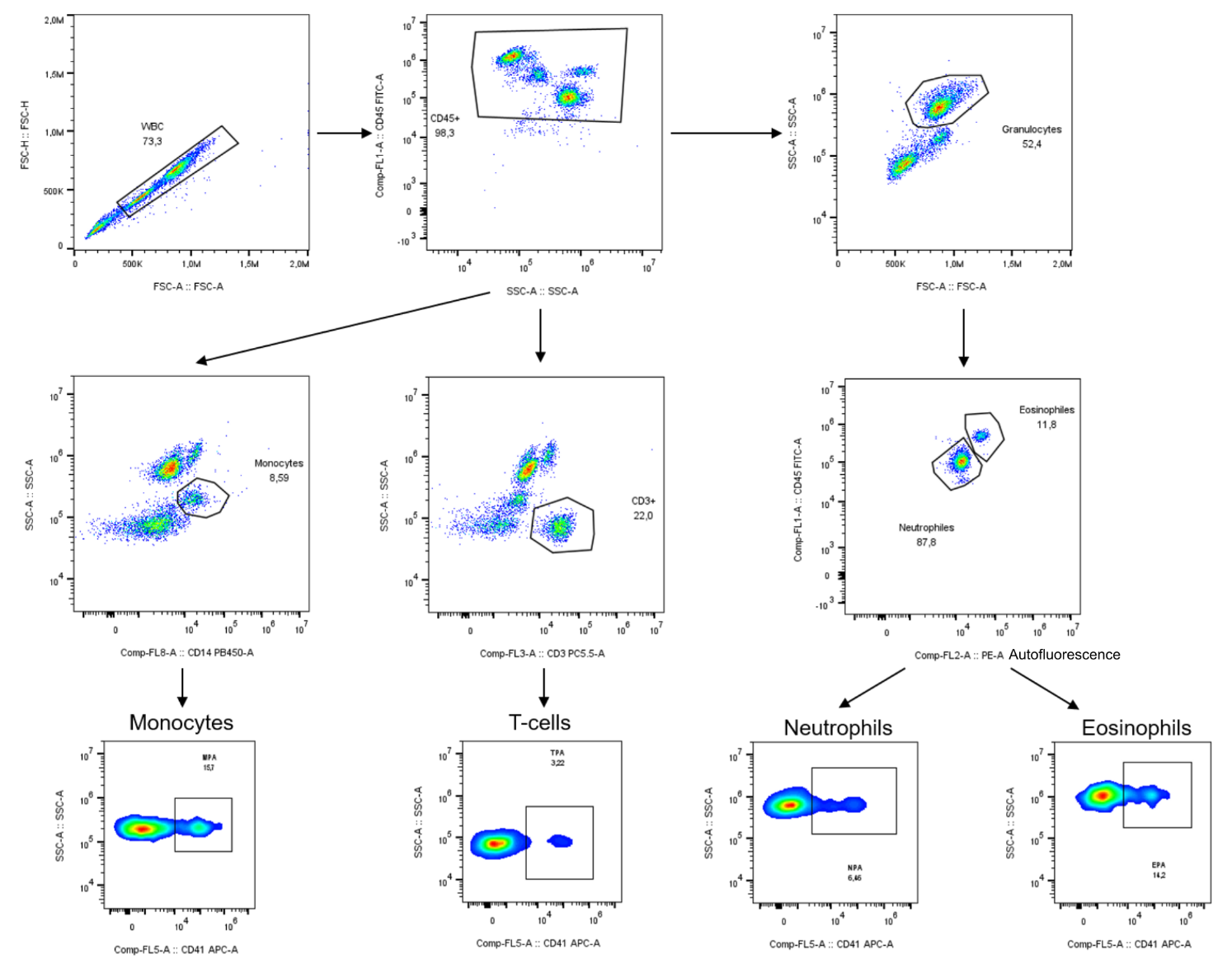


**Supplementary Figure S1:** **Gating strategy to identify leukocyte subsets in whole blood and to analyze CD41^+^ leukocytes.** CD45**^+^** white blood cells (WBC) were identified upon size by forward scatter (FSC) and CD45 expression. Subpopulations were classified using side scatter (SSC), specific markers and autofluorescence characteristics: CD14 high monocytes (CD45^+^CD14^+^), T-cells (CD45^+^CD3^+^), neutrophils (CD45^+^, SSC^high^), and eosinophils (CD45^+^, SSC^high^, with characteristic autofluorescence in the PE-channel [1-3]). Monocyte-platelet aggregates (MPA), T-cell-platelet aggregates (TPA), neutrophil-platelet aggregates (NPA) and eosinophil-platelet aggregates (EPA) are defined as CD41^+^ subpopulation.


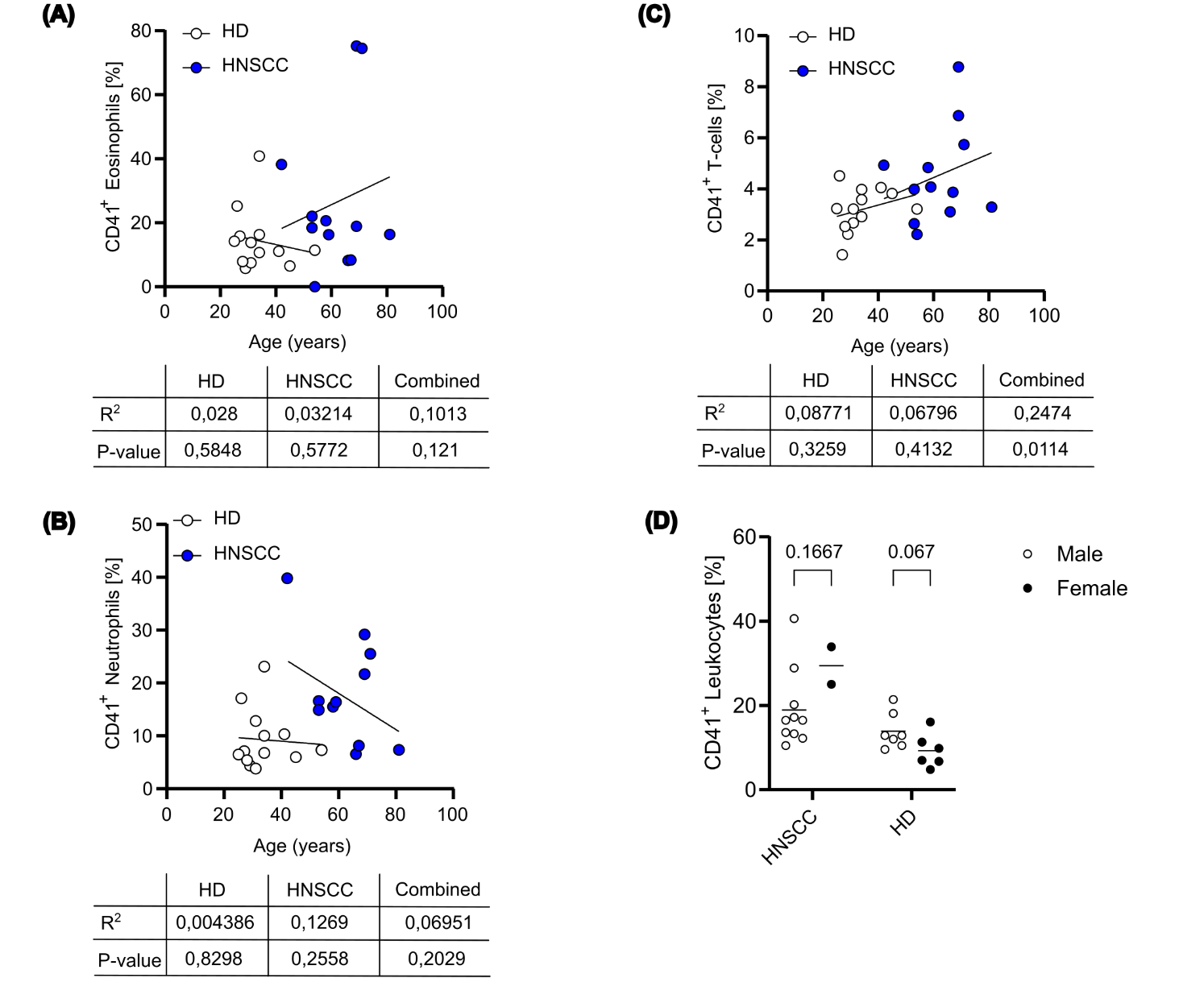


**Supplementary Figure S2: Relation of age and sex to PLA formation.** Correlation between CD41^+^ eosinophils **(A)**, CD41^+^ neutrophils **(B)**, CD41^+^ T-cells **(C)** and age were identified. Data were fitted with a simple linear regression, with the corresponding R^2^ and P-values indicated. **(D)** PLA percentage was compared between males and females of HD and HNSCC patients. Mann-Whitney test was used for comparison between healthy donors and HNSCC patients.


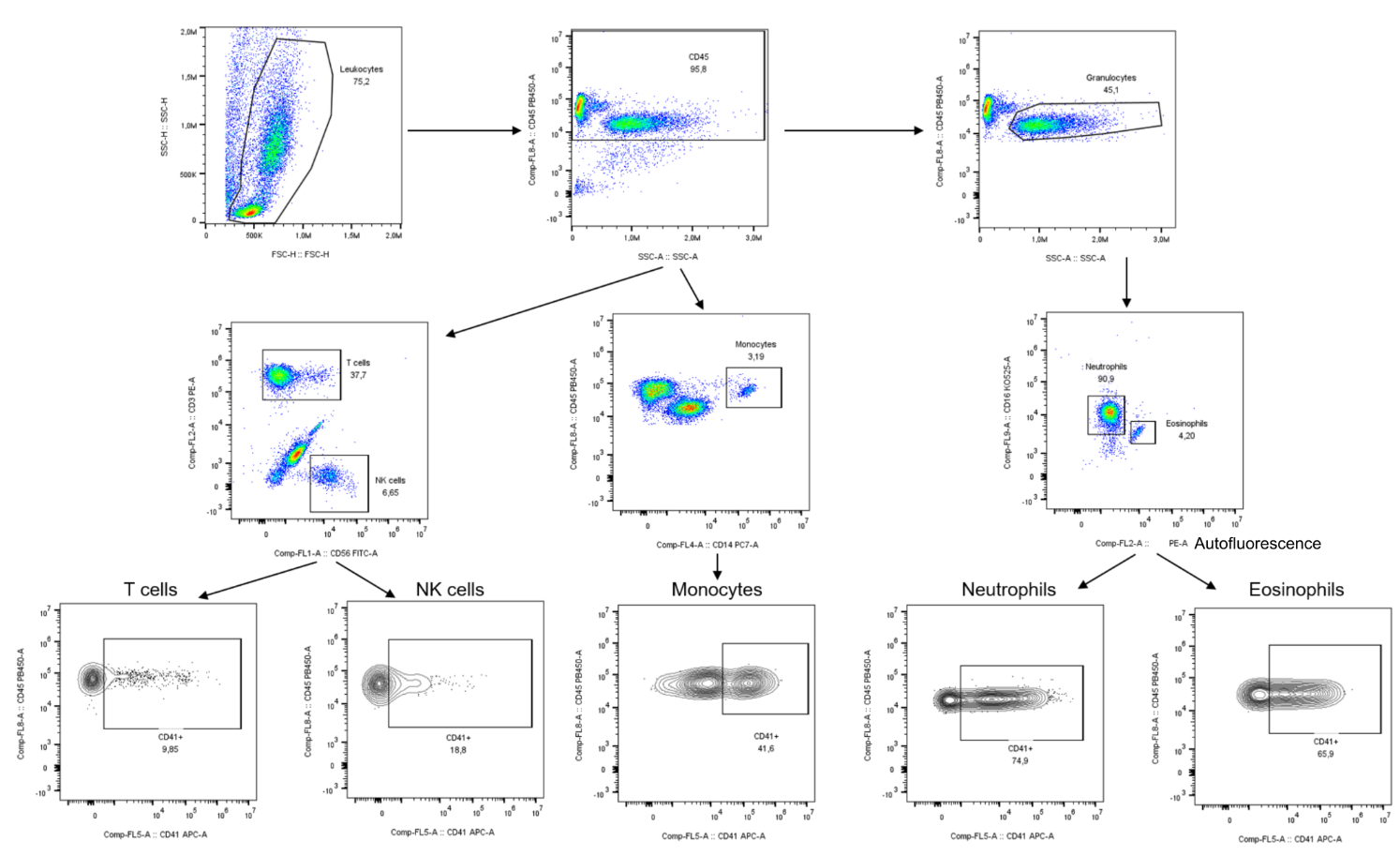


Supplementary Figure S3: Gating strategy to identify leukocyte subsets in *in vitro* co-culture model and to analyze CD41^+^ leukocytes. Leukocyte subpopulations were classified based on CD45 expression, side scatter (SSC) and autofluorescence characteristics: T cells (CD45^+^CD3^+^CD56^-^), NK cells (CD45^+^CD3^-^CD56^+^) and CD14 high monocytes (CD45^+^CD14^+^) were identified by their respective markers. Granulocytes were further divided into neutrophils (CD45^+^, SSC^high^,CD16^+^) and eosinophils (CD45^+^, SSC^high^, CD16^-^, with characteristic autofluorescence in the PE-channel [1-3]). Platelet-leukocyte aggregates are based on the CD41^+^ subpopulation.


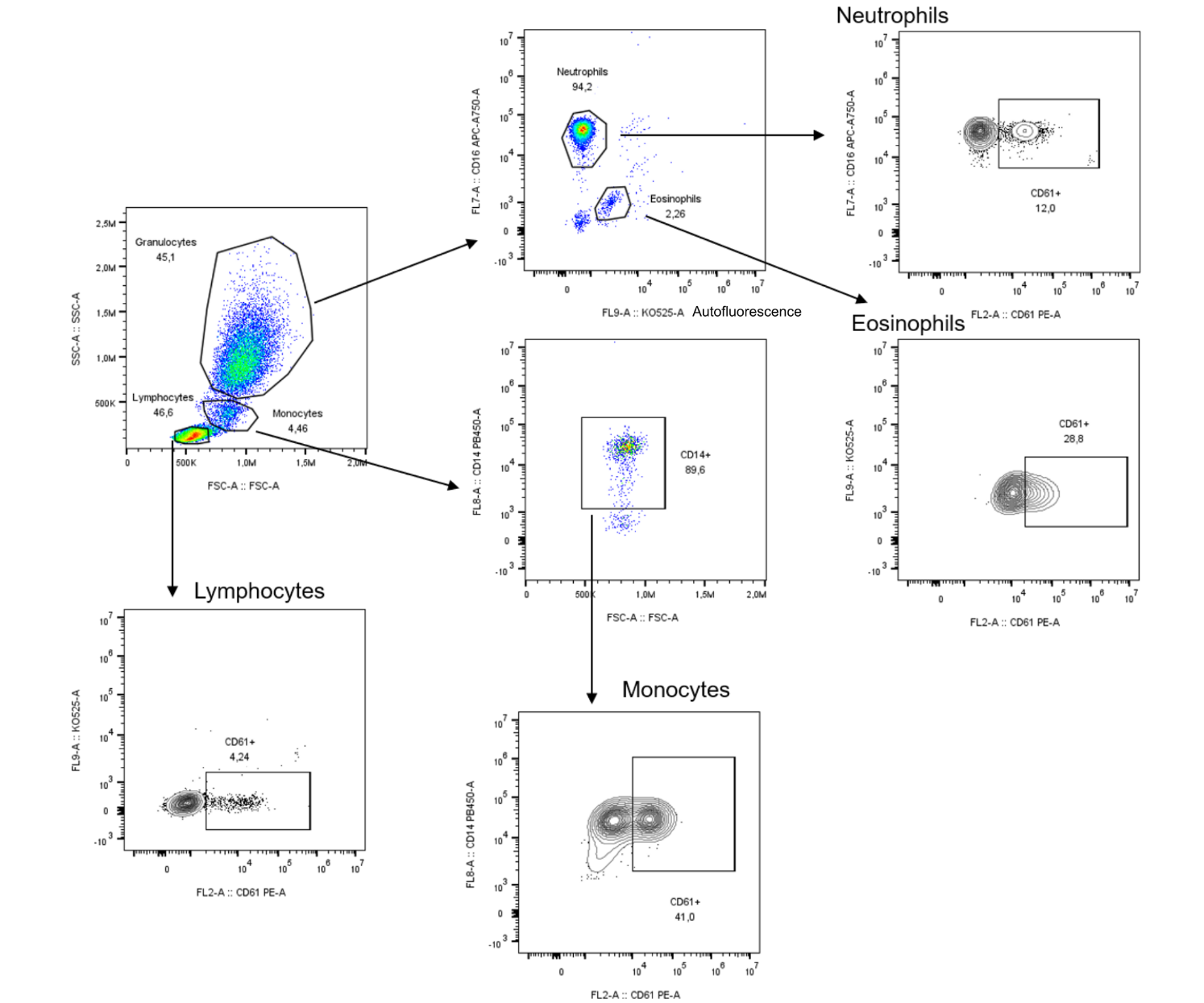


Supplementary Figure S4: Gating strategy to identify leukocyte subsets in *in vitro* blocking co-culture model and to analyze CD61^+^ leukocytes. Lymphocytes were identified based on their low forward scatter (FSC) and side scatter (SSC) properties. CD14 high monocytes were distinguished by intermediate FSC/SSC and CD14^+^ expression. Granulocytes were classified based on high FSC/SSC and further subdivided into neutrophils (CD16^+^) and eosinophils (CD16^-^). Eosinophils were specifically identified using their characteristic autofluorescence in the KO525 channel, allowing clear distinction from neutrophils [1-3]. Platelet-leukocyte aggregates were defined within the CD61+ subpopulation.


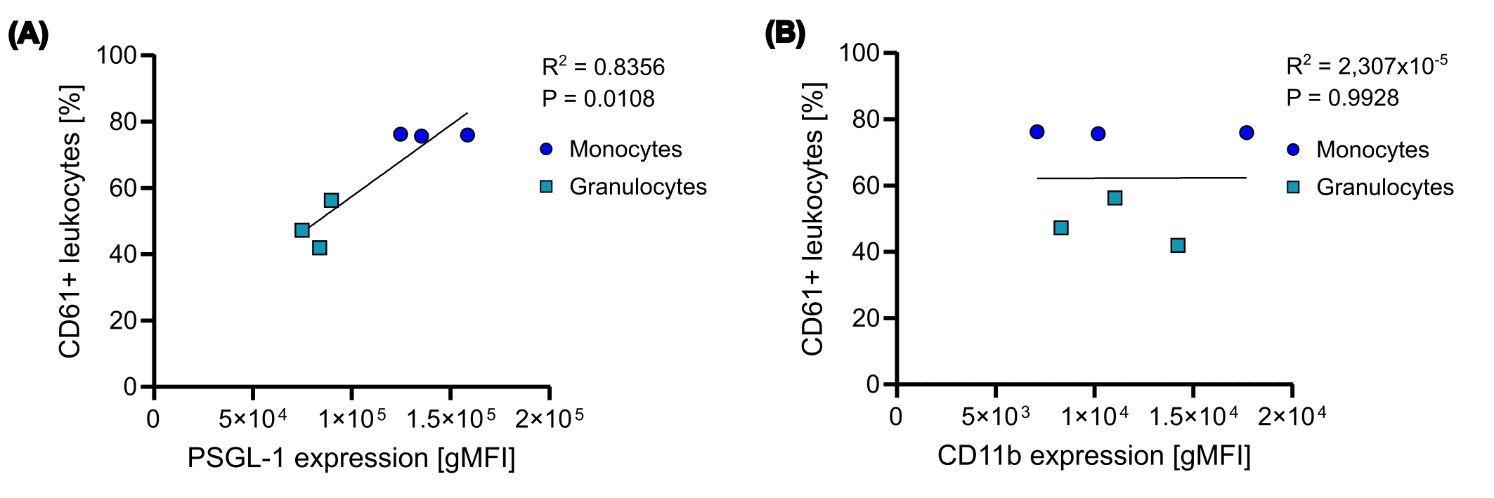


Supplementary Figure S5: Correlation of PSGL-1 and CD11b expression with platelet-monocyte and platelet-granulocyte aggregates. The percentages of CD61^+^ leukocytes were plotted against the expression levels of PSGL-1 (A) or CD11b (B). Data were fitted with a simple linear regression, with the corresponding R^2^ and p-value (P) indicated.


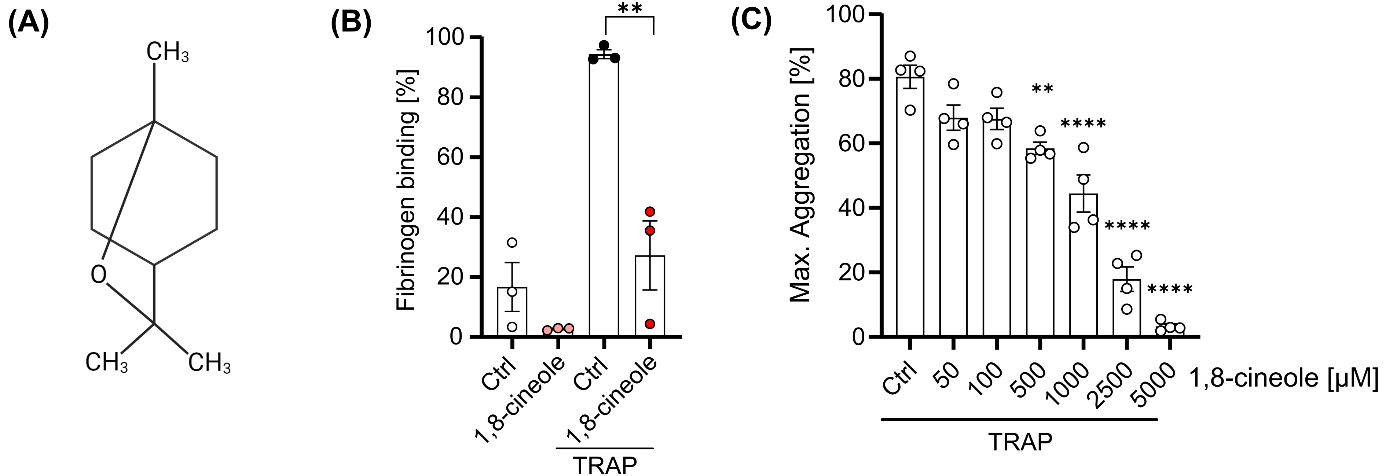


Supplementary Figure S6: 1,8-cineole dampened fibrinogen binding and platelet aggregation upon TRAP activation. (A) Chemical structure of 1,8-cineole. (B) Platelets were preincubated with and without 5 mM 1,8-cineole for 60 min prior to activation with TRAP for 10 min. Fibrinogen binding was measured by flow cytometry (n=3). (C) Platelets were preincubated with indicated concentrations of 1,8-cineole for 60 min before being activated with TRAP for 10 min. Maximal aggregation was assessed using aggregometry (n=4). Data are shown as mean ± SEM. **P<0.01. Unpaired Students’ t-tests were used for comparison between untreated and treated groups. Figure is created with BioRender.com


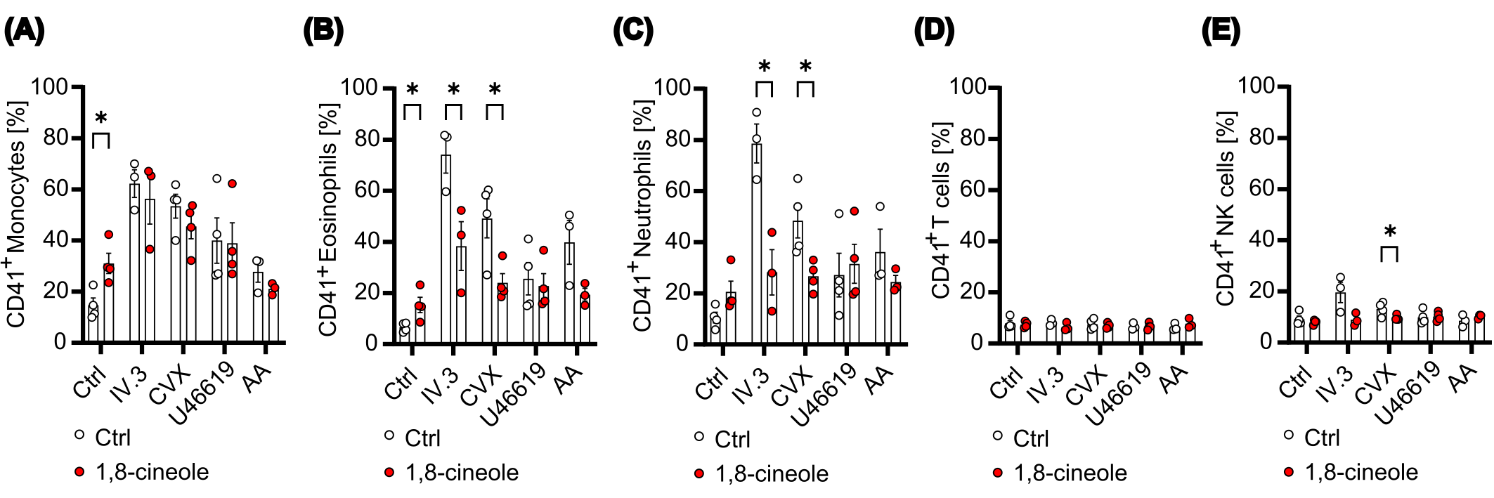


Supplementary Figure S7: Impact of 1,8-cineole on classical- and immunological-induced PLA formation. Platelets were pretreated with or without 1,8-cineole and subsequently activated with indicated agonists for 10 min, before being added to leukocytes. (A-E) PLA formation was analyzed after 15 min of co-culture by flow cytometry for different leukocyte subsets: (A) monocytes, (B) neutrophils, (C) eosinophils, (D) T cells and (E) NK cells (n=3). Data are presented as mean ± SEM. *P<0.05. Unpaired Students’ *t*-tests were used for comparison between untreated and treated groups.

Table 1: Characteristics of enrolled individuals

|  | HNSCC Patient (n=13) | HD (n=12) |
| --- | --- | --- |
| Age (mean ± SD) (years) | 61,83 ± 10,58 | 34,62 ± 8,94 |
| Sex (male:female) | 10:2 | 7:6 |

**References**

1. Ethier, C., P. Lacy, and F. Davoine, *Identification of Human Eosinophils in Whole Blood by Flow Cytometry*, in *Eosinophils: Methods and Protocols*, G.M. Walsh, Editor. 2014, Springer New York: New York, NY. p. 81-92.

2. Weihrauch, T., et al., *Protocol for autofluorescence-driven isolation of human peripheral blood eosinophils.* STAR Protocols, 2024. **5**(4): p. 103451.

3. Weil, G.J. and T.M. Chused, *Eosinophil Autofluorescence and its Use in Isolation and Analysis of Human Eosinophils Using Flow Microfluorometry.* Blood, 1981. **57**(6): p. 1099-1104.
